# Variation in Research Designs Used to Test the Effectiveness of Dissemination and Implementation Strategies: A Review

**DOI:** 10.3389/fpubh.2018.00032

**Published:** 2018-02-19

**Authors:** Stephanie Mazzucca, Rachel G. Tabak, Meagan Pilar, Alex T. Ramsey, Ana A. Baumann, Emily Kryzer, Ericka M. Lewis, Margaret Padek, Byron J. Powell, Ross C. Brownson

**Affiliations:** ^1^Prevention Research Center in St. Louis, Brown School, Washington University in St. Louis, St. Louis, MO, United States; ^2^Department of Psychiatry, Washington University School of Medicine, St. Louis, MO, United States; ^3^Brown School of Social Work, Washington University in St. Louis, St. Louis, MO, United States; ^4^School of Social Work, University of Maryland, Baltimore, MD, United States; ^5^Department of Health Policy and Management, Gillings School of Global Public Health, University of North Carolina at Chapel Hill, Chapel Hill, NC, United States; ^6^Department of Surgery, Alvin J. Siteman Cancer Center, Washington University School of Medicine, Washington University in St. Louis, St. Louis, MO, United States

**Keywords:** research study design, research methods, review, implementation research, dissemination research

## Abstract

**Background:**

The need for optimal study designs in dissemination and implementation (D&I) research is increasingly recognized. Despite the wide range of study designs available for D&I research, we lack understanding of the types of designs and methodologies that are routinely used in the field. This review assesses the designs and methodologies in recently proposed D&I studies and provides resources to guide design decisions.

**Methods:**

We reviewed 404 study protocols published in the journal *Implementation Science* from 2/2006 to 9/2017. Eligible studies tested the efficacy or effectiveness of D&I strategies (i.e., not effectiveness of the underlying clinical or public health intervention); had a comparison by group and/or time; and used ≥1 quantitative measure. Several design elements were extracted: design category (e.g., randomized); design type [e.g., cluster randomized controlled trial (RCT)]; data type (e.g., quantitative); D&I theoretical framework; levels of treatment assignment, intervention, and measurement; and country in which the research was conducted. Each protocol was double-coded, and discrepancies were resolved through discussion.

**Results:**

Of the 404 protocols reviewed, 212 (52%) studies tested one or more implementation strategy across 208 manuscripts, therefore meeting inclusion criteria. Of the included studies, 77% utilized randomized designs, primarily cluster RCTs. The use of alternative designs (e.g., stepped wedge) increased over time. Fewer studies were quasi-experimental (17%) or observational (6%). Many study design categories (e.g., controlled pre–post, matched pair cluster design) were represented by only one or two studies. Most articles proposed quantitative and qualitative methods (61%), with the remaining 39% proposing only quantitative. Half of protocols (52%) reported using a theoretical framework to guide the study. The four most frequently reported frameworks were Consolidated Framework for Implementing Research and RE-AIM (*n* = 16 each), followed by Promoting Action on Research Implementation in Health Services and Theoretical Domains Framework (*n* = 12 each).

**Conclusion:**

While several novel designs for D&I research have been proposed (e.g., stepped wedge, adaptive designs), the majority of the studies in our sample employed RCT designs. Alternative study designs are increasing in use but may be underutilized for a variety of reasons, including preference of funders or lack of awareness of these designs. Promisingly, the prevalent use of quantitative and qualitative methods together reflects methodological innovation in newer D&I research.

## Background

Dissemination and implementation (D&I) research is a relatively new scientific field that seeks to understand the scale up, spread, and sustainability of evidence-based interventions (EBIs) and practices for broad population health impact. D&I studies focus on effective strategies to enhance the speed of intervention implementation, quality of intervention delivery, and the extent to which the intervention reaches those it is intended to serve (1–4). D&I research is the final stage of the research to practice pipeline, and several characteristics of D&I studies differentiate them from efficacy and effectiveness studies. The exposures (the independent variables) in D&I studies are D&I strategies, whereas in efficacy and effectiveness studies, the exposures are the EBIs themselves (4). In D&I studies, outcomes are often related to the speed, quality, or reach of intervention implementation or delivery; these are often proximal outcomes, processes, and outputs of the service delivery system, and sometimes distal patient-level outcomes (1–4). As such, D&I studies are inherently multilevel, and accurate evaluation requires an understanding of the levels at which interventions are tested, implemented, and measured (5). D&I study outcomes are distinct from those in efficacy and effectiveness trials, which are related to changes in the target behaviors of end users or determinants of those behaviors (3). Due to the differences in D&I studies compared to efficacy and effectiveness studies of underlying interventions, the prioritization of study design considerations and study designs needed for D&I research are likely different than those of efficacy and effectiveness studies.

Traditional study designs such as randomized controlled trials (RCTs) can be ideal for testing the efficacy or effectiveness of interventions, given the ability to maximize internal validity. However, there has been concern that traditional designs may be ill-suited for D&I research, which requires a greater focus on (a) external validity; (b) implementation-related barriers and facilitators to routine use and sustainability of “effective” practices (6); (c) studying factors that lead to uptake of effective practices at the organizational level; and (d) capturing “moderating factors that limit robustness across settings, populations, and intervention staff,” including race/ethnicity, implementation setting, or geographic setting (7). Designs that enhance external validity allow us to better understand how interventions and implementation strategies work under realistic conditions rather than in highly controlled circumstances.

A number of alternative designs are available that give researchers flexibility and allow them to maximize external validity, match the research question of interest appropriately with the phase of D&I research (i.e., exploration, preparation, implementation, and sustainability) (4, 8, 9), and balance other trade-offs influencing the choice of design (10) (e.g., if randomization is appropriate, preference of stakeholders, etc.). If a randomized design is desired, it may be necessary to consider non-traditional ways to randomize, such as by time, to balance internal and external validity (4), and the practical, ethical, and pragmatic considerations that make some randomized designs less appealing in D&I research (4, 6, 9). For example, there is an ethical justification for designs that allow all stakeholders to receive an EBI and/or D&I strategy that is thought to be efficacious (11), since D&I studies focus on changes in organizations and communities led by stakeholders in these settings who often have more at stake than researchers (9). If a randomized design is not appropriate, other design features can be used to increase internal validity, such as multiple data collection points before and after the EBI is implemented (9). The evaluation of D&I strategies focuses on the process of implementation and stakeholders’ perceptions of this process (12, 13), and the choice of study design depends in part on the preferences of these stakeholders. Thus, a variety of designs that accommodate these considerations will likely be necessary to respond to calls from the National Academy of Medicine (formerly the Institute of Medicine) and numerous other organizations to accelerate the reach of EBIs and close gaps in the quality of health care and public health efforts (14–20).

Some of the alternative designs that are particularly suited to D&I research include interrupted time series, factorial designs, and rollout designs. An interrupted time series (21), in which multiple observations are taken before and after implementing an EBI, might be ideal when selecting the most cost-effective EBI and implementation strategy in the exploration phase. A factorial design, in which the combination of multiple D&I strategies are tested, could be more useful when testing the effectiveness of several different implementation strategies alone or in combination in the implementation phase. Adaptive designs are those in which study characteristics (e.g., implementation strategy type or mode) change throughout the study and may be useful when determining the sequence and combination of implementation strategies (22). Additionally, rollout designs (9), in which the timing of EBI implementation is randomly assigned, are a broad category of designs that include stepped wedge designs (23), where sites continue with usual practice until randomly assigned to transition to the EBI implementation for a defined period. These rollout designs may be more appealing or seen as more ethical to stakeholders than a cluster randomized trial with a no treatment control group, since all participants receive the D&I strategy and intervention packages at some point during the study period (24). There are many considerations that contribute to the choice of design, and assessment of the designs currently being used in D&I research is needed so that future implementation efforts may better account for these differences as well as the contextual factors and multiple levels involved in this field of study (25, 26).

This review was inspired by workgroup meetings supported by the United States (US) National Institutes of Health, “Advancing the Science of Dissemination and Implementation,” which focused on research designs for D&I research. The workgroup described 27 available designs (27), which have been categorized by Brown and colleagues into three types: within-site designs; between-site designs; and within- and between-site designs (9). Despite the increasing recognition of the need for optimal study designs in D&I research (4, 6), we lack data on the types of research designs and methodologies that are routinely used in D&I research. Therefore, we aimed to fill this gap by exploring the range of designs and methodologies used in recently proposed D&I studies testing implementation strategies. Our goals were to assess variation in designs and methodologies used, potentially categorize innovative design approaches, and identify gaps upon which future studies can build.

## Methods

**Figure 1:**
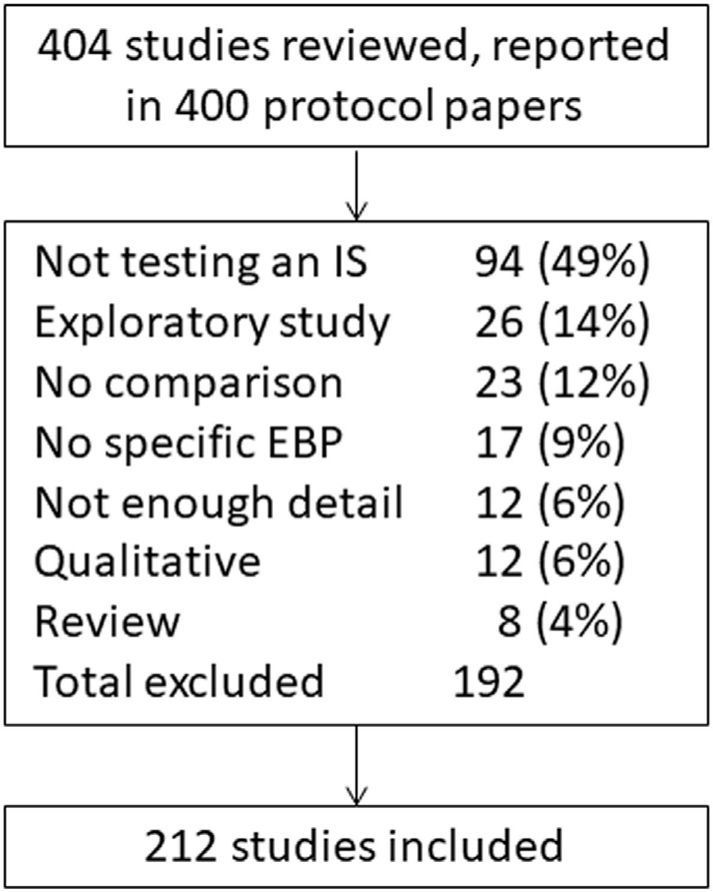
Overview of review process.

Study protocols published in *Implementation Science* from 2/22/2006 to 9/7/2017 (*n* = 400 manuscripts) were screened for eligibility (Figure 1). Manuscripts reporting study protocols typically provide detailed information about the study design and levels of intervention implementation and measurement; as such, this review included only study protocols to assess these factors across studies. To identify studies that were likely to use a variety of innovative methods, our search focused on *Implementation Science*, one of the top journals dedicated to publishing D&I research (28) that also has a specific designation for protocols. In addition, the journal has a focus on publishing “articles that present novel methods (particularly those have a theoretical basis) for studying implementation processes and interventions” (29).

Two of the included protocol manuscripts provided the descriptions of three studies each, resulting in 404 studies reviewed. To be included for full review, studies needed to test the efficacy or effectiveness of D&I strategies using some sort of comparison design. Studies were excluded if they were not testing a D&I strategy, if they were only testing the efficacy or effectiveness of a clinical or public health intervention itself, if they were purely qualitative, or if they did not include a comparison involving the D&I strategy (e.g., by group or time). D&I strategies are processes and activities used to communicate information about interventions and to integrate them into usual care and community settings (4, 27, 30–33). We used previous work by Powell and colleagues to categorize implementation strategies (27) to represent both D&I strategies within this review, since there has been more work done to articulate and categorize implementation strategies compared with dissemination strategies and there is likely a high amount of overlap between the strategies for each category of research (34).

A data extraction template was used to code the following design elements: design category (e.g., randomized, observational); design type (e.g., cluster RCT, pre–post no control); data collection with quantitative only or a combination of quantitative and qualitative methods; conceptual/theoretical framework used; levels of assignment, intervention, and measurement (30, 35); and country in which the research was conducted. Reviewers coded design types exactly as they were presented by study authors to capture the variety of terms used for study designs; for example, the same design was referred to as “interrupted time series with no controls” and “pre–post, interrupted time series” in different studies. Hybrid designs, those blending elements of effectiveness and implementation studies in one trial (6), were not specifically coded so that manuscripts published before this term was introduced could be included. Studies that were labeled as a hybrid study by authors were coded according to the design by which authors tested the implementation strategy. Levels of assignment, intervention, or measurement were coded as individual client or provider; groups/teams of clients or providers (e.g., a surgical unit within a hospital); organization (e.g., local health department); or larger system environment (e.g., province) (35). Each protocol was double-coded, and the few discrepancies were resolved through discussion with the study team.

**Figure 2:**
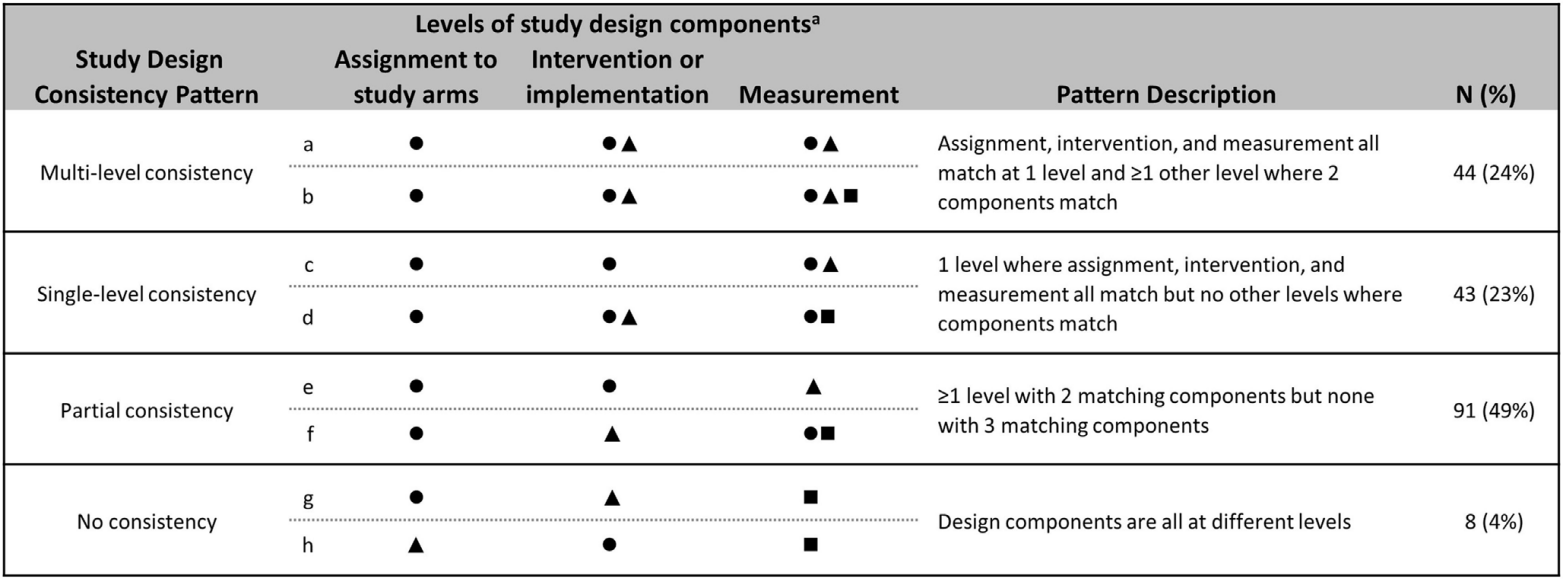
Consistency across levels of assignment, intervention, and measurement. Patterns of consistency across study design components are illustrated with eight example studies. Design components included are assignment (i.e., random or non-random allocation to study arms), intervention and/or implementation efforts, and measurement. Studies were grouped by patterns of consistency of levels across design components. The number and proportion of reviewed studies that fall into each consistency pattern are included. ^a^Symbols indicate the presence of a design component at a given level. Levels are defined as: ⚫ Organization, e.g., hospital, school. ▲ Provider, e.g., doctor, teacher. ■ Client, e.g., patient, student.

Some have suggested that it is most appropriate to assign to a treatment arm and measure at the level of implementation (i.e., at the level where the full impact of the strategy is designed to occur) (9, 36). Therefore, studies were grouped according to the extent to which there was consistency between design components: the levels of assignment, intervention, and measurement (Figure 2). *No consistency* occurred when design components were all at different levels. *Partial consistency* occurred when there was at least one level with two matching components, but none with three matching components. *Single-level consistency* occurred when intervention components and measurement were at the level of assignment. *Multilevel consistency* occurred when intervention components and measurement were at the level of assignment and there was at least one additional level with matching intervention components and measurement.

## Results

### Study Designs

Of the 404 studies screened, 212 (52%) tested one or more implementation strategy (Figure 1). The most common reasons for exclusion were the studies that did not test an implementation strategy (*n* = 94, 49%), were an exploratory study (*n* = 26, 14%), or the studies that did not have a comparison (*n* = 23, 12%). Of the included studies, 164 (77%) utilized randomized designs, primarily cluster randomized trials (*n* = 103, 49%), RCTs (*n* = 28, 13%), or stepped wedge cluster randomized trials (*n* = 16, 8%, Table 1). Only 35 studies (17%) were quasi-experimental and fewer (*n* = 13, 6%) were observational. One paper (37) that reported three studies included in this review contained very little information in the manuscript on study design; these studies were determined to be randomized trials according to context provided in the paper and group consensus. There was considerable variation in the way authors described their study designs. For example, “pre–post with controls” and “cluster controlled pre–post” both referred to the same methodological approach. These subtle differences in study design are likely important and reflect differences in the population, data type, and contextual influences available to the study authors. Complete coding for each study is available (Data Sheet S1 in Supplementary Material).

**Table 1:** Frequency of design described across the protocols reviewed.

	*n*	%
**Individual-level designs**	**30**	**14.2**
Randomized controlled trial (RCT)	29	13.7
Randomized factorial design	1	0.5
**Within-site designs**	**32**	**11.3**
*Observational*		
Cohort	4	1.9
Multiple case study	2	0.9
Retrospective case study	2	0.9
*Quasi-experimental*		
Pre–post without controls	13	6.1
Interrupted time series with no controls^a^	6	2.8
Multiple baseline design	2	0.9
Cross sectional	1	0.5
Phased implementation	1	0.5
*Random assignment to treatment arms*		
Randomized crossover	1	0.5
**Between-site designs**	**132**	**62.3**
*Random assignment to treatment arms*		
Cluster randomized trial^b^	107	50.5
Cluster randomized factorial trial	4	1.9
2 × 2 Factorial randomized control trial	1	0.5
Cluster randomized control trial-post-test only	1	0.5
Cluster randomized SMART implementation trial	1	0.5
Partial factorial cluster randomized trial	1	0.5
Single factorial design	1	0.5
*Non-random assignment to treatment arms*		
Pre–post with controls^c^	8	3.8
Comparative case study	3	1.4
Interrupted time series with controls	5	2.4
**Within- and between-site designs**	**18**	**8.5**
*Random assignment to treatment arms*		
Non-randomized stepped wedge trial	1	0.5
*Non-random assignment to treatment arms*		
Stepped wedge cluster RCT	16	7.5
Dynamic RCT	1	0.5

Total	212	100

*^a^Includes studies labeled as pre–post, interrupted time series*.

*^b^Includes studies labeled as cluster randomized comparative effectiveness trial*.

*^c^Includes studies labeled as cluster controlled pre–post and matched pair cluster design*.

There was a notable increase in the use of alternative designs over time. For example, stepped wedge designs were not used before 2011, but were proposed in at least four studies per year in 2014–2016. Conversely, there was a decrease in the reliance on individual-level RCTs. Between 2006 and 2012, RCTs represented 20% of all studies, whereas they only represented 8% of studies between 2013 and 2017. Additionally, researchers are utilizing a wider range of designs. From 2006 to 2012, there was an average of four types of designs used per year, which increased to 8.8 per year between 2013 and 2017.

### Levels of Assignment, Intervention, and Measurement

#### Assignment

For most studies (*n* = 124, 67%), the intervention was assigned at the level of the organization. Twenty-three studies (12%) used assignment at the level of the individual provider, and the remainder of the studies (*n* = 39, 21%) reported some combination of individual client, individual provider, group/team provider, and organization.

#### Intervention

Interventions were most commonly targeted at the individual provider (*n* = 51, 27%); the individual provider and the organization (*n* = 29, 16%); the organization alone (*n* = 23, 12%); or both the individual provider and client (*n* = 20, 11%). There were several studies that targeted clients, providers, and the organization (*n* = 14, 8%); individual providers and groups/teams of providers (*n* = 14, 8%), or groups/teams of providers (*n* = 11, 6%). The remaining studies targeted a variety of levels, for example, clients and larger system environments.

#### Measurement

Studies most frequently (*n* = 45, 24%) measured outcomes at the individual provider and client levels with fewer studies measuring at the level of the client, provider, and organization (*n* = 32, 17%) or clients alone (*n* = 21, 11%). Several studies also conducted measurement at the level of the organization (*n* = 18, 10%) and the level of the individual provider (*n* = 10, 9%). The remaining studies measured across other combinations, groups/teams of providers, or larger system environments.

#### Consistency across Levels

Consistency of assignment levels with intervention levels and assignment levels with measurement levels were comparable, with 113 (61%) of studies having intervention targets that matched the level of assignment and 120 (65%) having measures that matched the level of assignment. Those studies that were not consistent between assignment and intervention (*n* = 73, 39%) were predominately the studies that were assignment at the organization level, but intervened at the provider level. Similarly, those that were inconsistent between assignment and measurement levels (*n* = 66, 35%) were those that were assignment at the organization level and were measured at the individual client or provider levels.

The consistency between levels of intervention and measurement was more variable. Most studies had one level of intervention (*n* = 56, 30%) or multiple levels of intervention (*n* = 55, 30%), which had corresponding levels of measurement. Thirty-five studies (19%) had some overlap between intervention and measurement levels, for example, studies that intervened at the individual provider and organizational level, but measured at the individual client and provider levels. Forty studies (22%) had no consistency between intervention and measurement levels, for example, studies that intervened at the provider level, but measured at the client level. Comparing across all three levels, 44 (24%) studies had multilevel consistency between the level of assignment, intervention, and measurement, while 43 (23%) were consistent across a single level (Figure 2). Ninety-one studies (49%) were partially consistent, for example, assignment occurred at the level of the individual provider, intervention occurred at the level of the individual provider, and measures were taken at the level of the individual client.

### D&I Models, Theories, and Frameworks

Included protocols utilized a wide range of D&I conceptual frameworks. One hundred and eleven (52%) of the studies reported using a D&I model, and there were a variety of models used. The Consolidated Framework for Implementing Research (38) and RE-AIM (39) models were the most commonly reported frameworks (*n* = 16 studies each). Promoting Action on Research Implementation in Health Services (40, 41) and the Theoretical Domains Framework (42) were each reported by 12 studies. Additional models that were used by multiple studies included diffusion of innovations (43) (*n* = 8) and the exploration, preparation, implementation, and sustainment model (EPIS, *n* = 5) (8). Seven models were each reportedly used in two or three studies: Grol and Wensing’s implementation of change model (44); UK MRC Complex Interventions Framework (45); Normalization Process Theory (46); Chronic Care Model (47); Dynamic Sustainability Framework (1); Greenhalgh’s Model of Diffusion of Innovation in Health Organizations (48); and the Ottawa Model of Research Use (49). The remaining three models appearing only once in the sample.

### Additional Study Characteristics: Data Type, Study Location, and Funding Sources

One hundred twenty-nine studies (61%) used some combination of quantitative and qualitative data collection methods, and (since we excluded qualitative only studies) the remaining 39% (*n* = 83) utilized only quantitative methods. The majority of studies were conducted in the US (*n* = 69, 33%) or Canada (*n* = 45, 21%). There were 21 (13%) studies from Australia and 24 studies (11%) from the Netherlands. The remaining studies took place across Europe, Africa, and Asia. When considering funding sources, 183 (86%) of studies relied on regional or national agency contributions. Twenty-eight (13%) studies were funded by a foundation or internal funding, and 18 (8%) studies were funded by a regional, national, or agency, and four (2%) were funded by industry. Several studies were funded by multiple types of funding, and as such, one study may be represented in more than one of these categories.

## Discussion

The current review found that of the included D&I studies from the protocol papers published in the journal *Implementation Science*, most are using cluster randomized trials or RCTs, although the use of RCTs has decreased. Though a number of other designs have been proposed to conduct D&I research (4, 50), these alternative designs may be under-represented in the current findings, and RCTs still predominate D&I literature (17). This is particularly noteworthy given the review included only protocol papers from the journal *Implementation Science*, which is likely more “open” to new/other types of D&I designs than other scientific journals. D&I studies are also being published in other journals, which may have an even lower rate of alternate design types. However, this field is still relatively new, and it may take time to see a more balanced distribution of study designs appear within peer-reviewed literature.

The increase in the variety of study designs used over time indicates that researchers are using alternative designs more frequently to answer different D&I research questions. As described by Aarons and colleagues, these questions take place across different phases of D&I research that include *exploration* to determine which EBI(s) to implement, *adoption/preparation* to understand factors related to the decision to implement an EBI, *implementation* to identify effective D&I strategies for improving program fidelity, and *sustainment* to examine strategies that promote maintained delivery or use of an EBI (8). Some designs may be more suited to answer particular research questions within each phase. For example, a comparative case study design is appropriate to identify a potentially effective implementation strategy to test in future research (51), while a cluster randomized stepped wedge design may be more appropriate when testing the effectiveness and sustainability of an implementation strategy (52). We could not code for this within our sample, as it is not always specified which phase researchers consider their research questions, but it is possible this is a factor in deciding which design to use.

Given the benefits of using a theory or framework to guide D&I research (53–57), it is surprising that the current review identified only 111 (52%) studies that described such grounding. Other reviews have also found low prevalence of theory and framework use (58–60), even though resources exist to help D&I researchers search for and identify appropriate theories or frameworks to guide their studies (61, 62). These studies may have a theoretical underpinning that was not articulated in the protocol. However, there is a need for wider use and reporting of theory and frameworks used, as they are known to increase the effectiveness of an implementation strategy (63), to understand the mechanism by which a program acts, and to promote replicability of studies.

Despite the significant benefits randomized trials can provide (i.e., internal validity), it is possible that their use may reduce external validity (64). Less traditional methods (e.g., multiple baseline design, phased implementation), which appear to be underutilized, provide enhanced flexibility and capacity to incorporate local context; these types of designs may additionally present more feasible options. Additionally, methods such as systems science and network analysis were not identified in the current review, but are growing in popularity in D&I research (65). However, it is possible that our inclusion criteria, particularly the requirement of a comparison group, may have excluded such methods.

**Figure 3:**
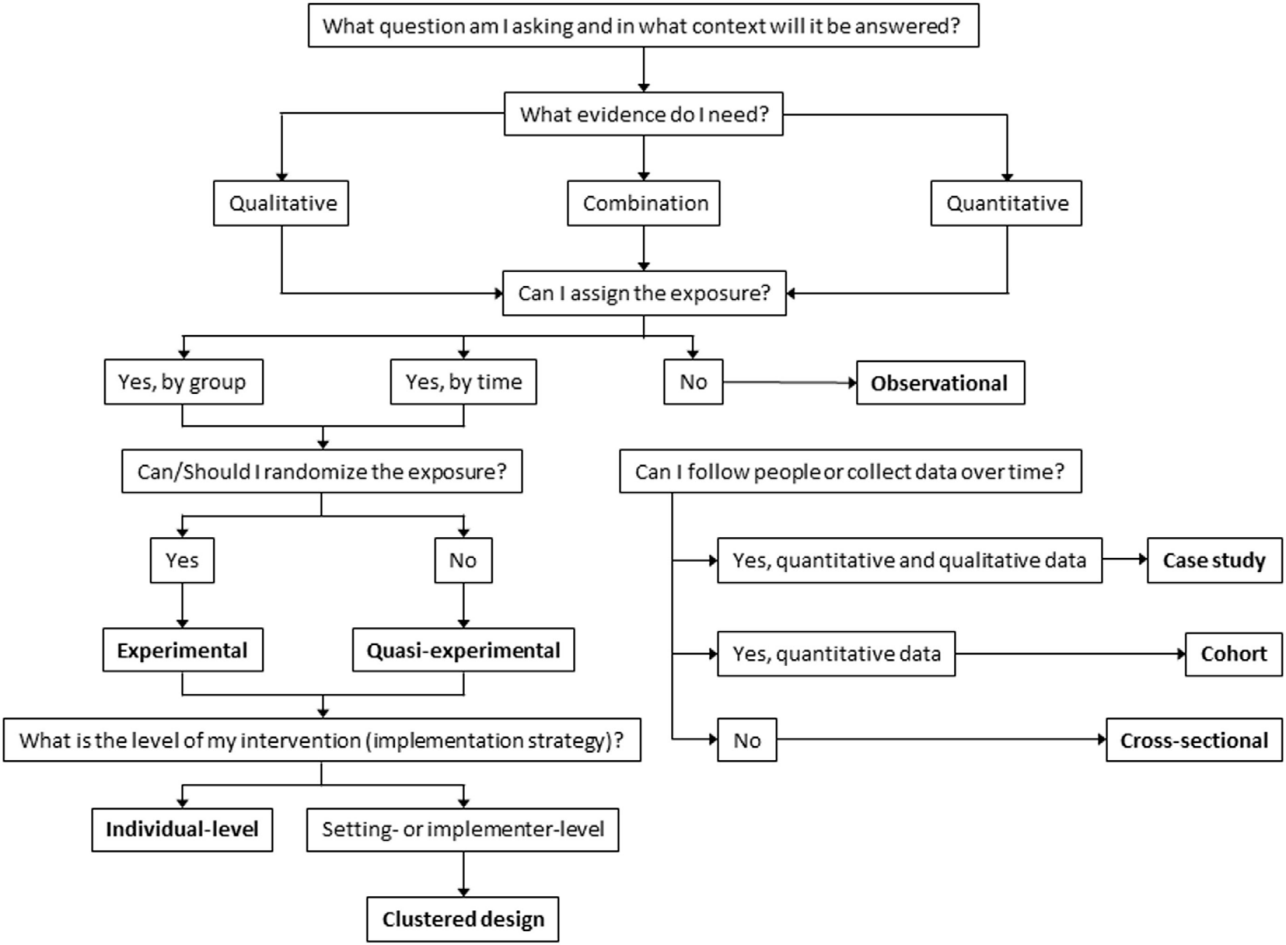
Decision tree for dissemination and implementation study designs.

While there has been an increase in the use of alternative designs, many researchers continue to rely on more traditional designs, such as RCTs, similar to a prior review of implementation studies specific to child welfare and mental health (30). There are likely many reasons researchers continue to utilize RCTs, including those designing and evaluating studies may perceive these as the best way to minimize selection bias. It is possible that our findings represent a dissemination issue, in that the use of alternate designs is gaining speed, but has been slow to spread through this newly developing field. To facilitate the spread of different and perhaps more appropriate designs and to assist investigators developing D&I studies, we have provided a guide for researchers making decisions about their study designs (Figure 3). This decision process begins with defining a research question (53–55), which determines whether the data needed should be quantitative, qualitative, or mixed. Once the research question and type of data are determined, it is important to consider whether it is possible and ethical to assign exposure and if the exposure can be assigned by group or by time. In the current review, the majority of studies reviewed included assigning exposures (*n* = 186, 88%). If assigning exposure randomly is ethical and practical, the study can be either experimental or if not, quasi-experimental; in the current review, 164 (77%) and 35 (17%) of included studies were randomized and quasi-experimental, respectively.

If randomization is not possible, then there are alternate ways to enhance the rigor of a design. For example, group equivalence at pre-test can be achieved by design factors such as matching or using matched controls (66). Other options to strengthen internal validity include multiple pre- and post-tests and/or removed then repeated interventions (9, 17, 50). In these types of studies, units can be randomized to different time periods (rather than only to groups), such as with stepped wedge designs. This helps account for time-related (e.g., history) threats to internal validity, etc., reducing threats to both internal and external validity (17, 23, 24). When assignment of exposure is possible, it is also important to consider the level at which exposure can/will be assigned (e.g., individual, organizational) and to address any clustering effect this might create through design, measurement, and analysis. Specific alternative designs do not appear in the figure; instead, opportunities for alternative designs exist within each category (e.g., randomize by time vs. condition).

Another alternative design when exposure is not ethical or possible is the observational design (67). The current review identified few studies using observational designs (*n* = 13, 6%). It is possible that our inclusion criteria may have led to this under-representation of observational designs, particularly cross sectional. Observational designs can vary considerably depending on whether data can be collected over time (i.e., longitudinal) or at only one time (i.e., cross sectional). It might be possible to enhance the evaluative power of an observational study if data collection can be timed around implementation of an intervention to create a natural experiment. Observational designs might also be useful in pre-intervention phases, identifying prevalence rates, potential intervention points, hypothesized causal pathways, potential mediators, and acceptable implementation strategies (9, 67). The rigor of these studies can be enhanced with data collection at more time points, and the internal validity can be improved if measures with more reliability and validity evidence are used.

There are issues that cut across all of these decisions about study designs. It is beyond the scope of this paper to discuss all the potential decisions that might arise in study design, but three are of particular importance: context, study level, and use of a theory or framework. Context is the setting in which practice takes place and is particularly important in D&I research (68). Whether study sites are selected to represent a range of different organizations with respect to cultures, climates, readiness, or just selecting the sites that are most “ready” or amenable to the implementation effort is an important decision point with implications for interpretation of findings. Regardless of the decision around the study design, it is important that consideration of context be explicitly incorporated into the study, such as in site selection, as it can have important implications on whether an intervention is implemented properly and therefore can have its intended effects. Determining the level for assignment, intervention, and measurement, all have important implications (e.g., in the school setting: individual students, classrooms, schools, school districts). Within the coding scheme used for this review, it was sometimes difficult to identify these characteristics of studies, possibly because of differences across substantive areas. With the low use of theory in the studies for this review, there is an opportunity to strengthen future research with the use of theory that guides implementation and measurement and is articulated. Better reporting of study characteristics can promote replicability and translation of knowledge across disciplines.

Analytical methods may be utilized to account for these decisions (e.g., the use of multilevel modeling). Where possible researchers should be consistent in the levels at which they assign, intervene, and measure effects. Though this does not prevent bias, which can still exist even with consistency, it lessens the chance. These decisions also have important implications for sample size and statistical power (i.e., unlike in a clinical trial, where the sample may be at the level of the individual, D&I studies often require that units be the cluster organization, hospital, school, agency level) as well as analysis; when clustering is present, appropriate statistical measures must be employed.

Several issues in D&I research should influence the design choice. For example, if the intervention evidence is sound, it may not be necessary to re-establish effectiveness; rather, one may be more interested in tracking the fidelity of implementation. This often implies the need for knowledge about organizational factors, including culture, climate, and readiness. In addition, measurement is important to consider. Whether or not measures exist to assess the factors in question, including the psychometric and pragmatic properties of these measures (69, 70), will inform design decisions (71, 72). The choice if a D&I design involves a series of trade-offs including some that are not addressed here, and these often balance scientific rigor with real-world circumstances (10). Specific examples of study designs proposed within this sample are available in Data Sheet S1 in Supplementary Material. Also, several examples have been presented in Data Sheet S2 in Supplementary Material based on the decision tree that detail some of these considerations, and Data Sheet S3 in Supplementary Material presents a compilation of resources available to support design choice.

This study has limitations worth noting. The first is that only protocol papers from one journal were included, and our sample may not be generalizable to all D&I research published in other journals or outside of a study protocol format. However, *Implementation Science* is on the forefront of the emerging field and likely represents a broad spectrum of studies being conducted in D&I research. Additionally, purely qualitative studies were not included in this review, and we did not code for how qualitative and quantitative data were used within a study. Though few studies were excluded for this reason alone (*n* = 12), studies of this nature may demonstrate use of alternate study designs. Future research on the use of mixed methods within D&I work is needed to understand how types of mixed methods approaches are applied in D&I research (73). Another limitation of our sampling is our focus on research that is testing D&I strategies, thus leaving out a whole set of D&I studies that focus primarily on understanding the context including influences on professional and organizational behavior; these studies are often shorter in duration and likely from smaller grants, where investigators may not publish protocol papers. Further, our sample may have suffered from selection bias, as trials are most likely to be funded and to benefit from publishing a protocol paper. Thus, it might be expected that RCTs and cluster RCTs were common. We were also limited in coding what was presented in the protocol paper, and in some cases, during implementation of a study, some changes may be made that are not reported in the original protocol (e.g., addition of constructs from a different theory). Last, we did not code how the qualitative data were used within studies using both qualitative and quantitative data, i.e., parallel sequential or converted approaches (33).

In the face of national and international calls for accelerating the spread of EBIs, policies, and treatments, maximizing the utility of the results for D&I studies is essential. This includes findings with robust internal validity while maximizing external validity and those that are relevant to the variety of stakeholders involved in D&I research. Fortunately, the field has a suite of designs, including many alternatives to RCTs, which can help answer these calls.

## Conclusion

While alternatives to the RCT (e.g., stepped wedge, adaptive designs) were employed in several studies, our review suggests that funded D&I research has largely mirrored clinical effectiveness research by primarily relying upon cluster RCTs and RCTs. However, alternative designs that offer researchers flexibility based on the context of their research and can maximize external validity are becoming more common. While the use of design approaches using qualitative and quantitative data sources appears to be prevalent in D&I research, there is a need for more use and reporting of D&I theory to guide future studies.

## Author Contributions

All authors made substantial contributions to conception and design, acquisition of data, and interpretation of data; SM and RT were involved in drafting the manuscript; MP, AR, AB, EK, EL, MP, BP, and RB have been involved in revising the manuscript critically for important intellectual content. All authors have given final approval of the version to be submitted and agree to be accountable for all aspects of the work in ensuring that questions related to the accuracy or integrity of any part of the work are appropriately investigated and resolved.

## Conflict of Interest Statement

The authors declare that the research was conducted in the absence of any commercial or financial relationships that could be construed as a potential conflict of interest.
