# Supplementary material for: Variation in Research Designs Used to Test the Effectiveness of Dissemination and Implementation Strategies: A Review
Source: Front Public Health. 2018 Feb 19;6:32. doi: 10.3389/fpubh.2018.00032 (PMC5826311; doi:10.3389/fpubh.2018.00032)
Supplement: Supplementary file 1 [file Data_Sheet_1.docx]

| **Reference** | **Design Category** | **Design Type** | **Level for Assignment** | **Level for Intervention** | **Data type** | **Level for Measurement** | **D&I Framework** | **Country** | **Funding Source** |
| --- | --- | --- | --- | --- | --- | --- | --- | --- | --- |
| Aarons 2012^1^ | Randomized | Cluster Randomized Trial | Organization | Individual (provider), Group/Team (provider), Organization | Quantitative and Qualitative | Individual (client, provider), Group/Team (provider), Organization | EPIS/Dynamic Adaptation Process | USA | National Funding Agency |
| Aarons 2017^2^ | Randomized | Cluster Randomized Trial | Group/team (provider) | Individual (provider), Group/team (provider), Organization | Quantitative and Qualitative | Individual (provider), Group/team (provider), Organization | EPIS | USA | National Funding Agency |
| Acolet 2007^3^ | Randomized | Cluster Randomized Trial | Group/Team (provider) | Individual (provider), Group/Team (provider) | Quantitative | Individual (client), Group/Team (client, provider) | Organizational development cycle | England | Foundation |
| Agoritsas 2014^4^ | Randomized | Randomized Factorial Design | Individual (provider) | Individual (provider) | Quantitative | Individual (provider) | NR | Canada | National Funding Agency |
| Allen 2013^5^ | Randomized | Cluster Randomized Trial | Organization | Individual (provider), Group/Team (provider), Organization | Quantitative | Individual (provider), Group/Team (provider), Organization | Framework adapted from: Kramer, 2003, 2004, and 2013 | USA | National Funding Agency |
| Amemori 2011^6^ | Randomized | Cluster Randomized Trial | Organization | Individual (provider) | Quantitative | Individual (provider), Organization | Theoretical-Domains Framework | Finland | National Funding Agency, National Agency, Foundation |
| Anderson 2012^7^ | Randomized | Randomized Controlled Trial | Organization | Organization | Quantitative | Organization | NR | USA | National Funding Agency |
| Anrys 2016^8^ | Randomized | Cluster Randomized Trial | Organization | Individual (provider), Group/team (provider) | Quantitative and Qualitative | Individual (client), Group/team (provider) | NR | New Zealand | National Funding Agency |
| Aron 2014^9^ | Observational | Cohort | N/A | Individual (provider), Organization | Quantitative and Qualitative | Individual (client, provider), Organization | Model of Innovation Dissemination/Diffusion, Consolidated Framework for Implementation Research | USA | National Funding Agency |
| Bartlem 2013^10^ | Quasi | Multiple Baseline Design | N/A | Individual (provider) | Quantitative | Individual (client, provider) | NR | Australia | Regional Funding Agency |
| Bauer 2016^11^ | Randomized | Stepped Wedge Cluster Randomized Controlled Trial | Organization | Individual (client, provider), Group/team (provider), Organization | Quantitative and Qualitative | Individual (client), Group/team (provider), Organization | i-PARIHS | USA | National Funding Agency |
| Beidas 2013^12^ | Observational | Pre-Post Without Controls | N/A | Individual (provider), Group/Team (provider) | Quantitative and Qualitative | Individual (provider), Organization | EPIS | USA | None |
| Blanchet 2016^13^ | Randomized | Stepped Wedge Cluster Randomized Controlled Trial | Larger System Environment | Individual (provider), Organization, Larger System Environment | Quantitative and Qualitative | Individual (provider), Organization | NR | Burkina Faso | National Agency |
| Boivin 2011^14^ | Randomized | Randomized Controlled Trial | Organization | Group/Team (provider) | Quantitative | Individual (provider), Group/Team (provider) | NR | Canada | National Funding Agency |
| Borgermans 2008^15^ | Randomized | Randomized Controlled Trial | Organization | Individual (provider) | Quantitative | Individual (client) | UK MRC framework for the development and evaluation of complex interventions for RCTs | Germany | National Agency |
| Borghi 2013^16^ | Quasi | Pre-Post With Controls | N/A | Organization | Quantitative | Individual (client, provider) | P4P | Tanzania | National Agency |
| Botti 2014^17^ | Quasi | Pre-Post Without Controls | N/A | Individual (provider), Organization | Quantitative | Individual (client, provider), Organization | PARiHS | Australia | National Funding Agency |
| Bower 2012^18^ | Randomized | Cluster Randomized Trial | Organization | Individual (client) | Quantitative and Qualitative | Individual (client) | NR | UK | National Funding Agency |
| Brouwers 2010^19^ | Randomized | Single Factorial Design | Individual (provider) | Individual (provider) | Quantitative | Individual (provider) | Instructional Systems Development framework, including the ADDIE | Canada | National Funding Agency |
| Brown 2014^20^ | Randomized | Stepped Wedge Cluster Randomized Controlled Trial | Organization | Individual (provider), Group/team (provider), Organization | Quantitative | Individual (provider), Organization | PRECEDE-PROCEED | Australia | National Funding Agency, National Agency? |
| Bucknall 2017^21^ | Randomized | Cluster Randomized Trial | Group/team (provider) | Individual (provider), Group/team (provider) | Quantitative and Qualitative | Individual (client, provider), Group/team (provider), Organization | PARiHS | Australia | National Funding Agency |
| Bunker 2012^22^ | Randomized | Cluster Randomized Trial | Organization | Individual (client) | Quantitative and Qualitative | Individual (client, provider) | NR | Australia | National Funding Agency |
| Campbell-Schere 2014^23^ | Randomized | Cluster Randomized Trial | Group/team (provider) | Individual (provider), Group/team (provider) | Quantitative and Qualitative | Individual (client, provider), Group/team (provider) | Theoretical Domains Framework, RE-AIM, Complex Innovation of Implementation | Canada | Regional Funding Agency |
| Charrois 2011^24^ | Randomized | Cluster Randomized Trial | Individual (client) | Individual (client, provider) | Quantitative and Qualitative | Individual (client, provider) | NR | Canada | National Funding Agency, Regional Funding Agency, Industry |
| Chinman 2017^25^ | Randomized | Cluster Randomized Trial | Organization | Individual (provider), Group/team (provider) | Quantitative and Qualitative | Individual (client, provider), Group/team (provider), Organization | i-PARIHS, RE-AIM | USA | National Funding Agency |
| Clemson 2017^26^ | Randomized | Cluster Randomized Trial | Organization | Individual (provider), Organization | Quantitative and Qualitative | Individual (client, provider), Organization | Knowledge-to-action framework (KAT), normalization process theory (NPT) | Australia | National Funding Agency |
| Cohen 2016^27^ | Observational | Cohort | N/A | Individual (provider) | Quantitative and Qualitative | Individual (client)?, Organization, Larger System Environment | Consolidated Framework for Implementation Research (CFIR), Practice Change Model (PCM) | USA | National Funding Agency |
| Cooper 2009^28^ | Randomized | Randomized Controlled Trial | Individual (client, provider) | Individual (client, provider) | Quantitative and Qualitative | Individual (client, provider) | NR | USA | National Funding Agency |
| Cooper 2013^29^ | Quasi | Pre-Post Without Controls | N/A | Individual (client, provider), Group/Team (provider), Organization | Quantitative and Qualitative | Individual (client, provider), Group/Team (provider), Organization | Merged framework of 3 models: Program change model (Simpson), CFIR (Damschroder), Program sustainability (Shediac-Rizkallah & Bone) | USA | National Funding Agency |
| Couturier 2015^30^ | Quasi | Pre-Post Without Controls | N/A | Individual (provider), Group/team (provider), Organization | Quantitative and Qualitative | Individual (provider), Organization | Consolidated Framework for Advancing Implementation Research (CFIR) | Canada | National Funding Agency |
| Cowan 2015^31^ | Randomized | Stepped Wedge Cluster Randomized Controlled Trial | Organization | Individual (provider), Group/team (provider), Organization | Quantitative and Qualitative | Individual (client), Organization | NR | Mozambique | National Funding Agency |
| Cucciare 2016^32^ | Randomized | Randomized Controlled Trial | Individual (provider) | Individual (provider) | Quantitative and Qualitative | Individual (provider, client), Organization | PARiHS | USA | National Funding Agency |
| Cully 2012^33^ | Randomized | Randomized Controlled Trial | Individual (client) | Individual (client, provider) | Quantitative and Qualitative | Individual (client, provider) | RE-AIM | USA | National Agency |
| Dainty 2011^34^ | Randomized | Randomized Controlled Trial | Organization | Individual (provider), Group/Team (provider), Organization | Quantitative | Individual (client) | NR | Canada | National Funding Agency, Foundation |
| Damschroder 2015^35^ | Quasi | Pre-Post Without Controls | N/A | Individual (client, provider), Organization | Quantitative and Qualitative | Individual (client, provider), Organization | Program change model (Simpson), CFIR, RE-AIM | USA | National Funding Agency |
| de Groot 2015^36^ | Randomized | Cluster Randomized Trial | Organization | Individual (provider), Organization | Quantitative and Qualitative | Individual (client, provider) | NR | Netherlands | National Funding Agency |
| de Lusignan 2009^37^ | Randomized | Cluster Randomized Trial | Organization | Organization | Quantitative and Qualitative | Individual (client, provider) | NR | England | Foundation |
| Dekker 2010^38^ | Randomized | Randomized Controlled Trial | Organization | Individual (client, provider) | Quantitative | Individual (client, provider) | NR | Netherlands | National Funding Agency |
| Del Cura-Gonzales 2016^39^ | Randomized | Cluster Randomized Trial | Group/team (provider) | Individual (provider), Group/team (provider) | Quantitative and Qualitative | Individual (client, provider), Group/team (provider) | NR | Spain | National Funding Agency |
| Deneckere 2012^40^ | Randomized | Cluster Randomized Control Trial-Post-Test Only | Individual (provider) | Individual (provider) | Quantitative | Individual (provider) | NR | Belgium, Ireland, Italy, and Portugal | Industry |
| DeVoe 2015^41^ | Randomized | Cluster Randomized Trial | Organization | Individual (provider), Organization | Quantitative and Qualitative | Individual (client, provider), Organization | RE-AIM | USA | National Funding Agency |
| Dorr 2015^42^ | Randomized | Cluster Randomized Trial | Organization | Individual (provider), Organization | Quantitative and Qualitative | Individual (client), Group/team (provider), Organization | NR | USA | Foundation |
| Dorsey 2013^43^ | Randomized | Randomized Controlled Trial | Individual (provider) | Individual (provider) | Quantitative and Qualitative | Individual (client, provider), Organization | NR | USA | National Funding Agency |
| Eccles 2007^44^ | Randomized | Randomized Controlled Trial | Individual (provider) | Individual (provider) | Quantitative | Individual (provider) | MRC and Theory of planned behavior | UK | National Funding Agency |
| Eiraldi 2014^45^ | Randomized | Cluster Randomized Trial | Organization | Group/Team (provider) | Quantitative and Qualitative | Individual (client), Organization | Public Health Model; Proctor Implementation Outcomes Taxonomy | USA | Internal |
| Eiraldi 2016^46^ | Randomized | Cluster Randomized Trial | Organization | Individual (provider), Organization | Quantitative and Qualitative | Individual (client, provider) | Interactive Systems Framework | USA | National Funding Agency |
| Elliot 2010^47^ | Observational | Cohort | Organization | Individual (client), Organization | Quantitative and Qualitative | Individual (client), Organization | RE-AIM; Framework for Effective Translation. Modified from Durlak and DuPre | USA | National Funding Agency |
| Emond 2015^48^ | Randomized | Stepped Wedge Cluster Randomized Controlled Trial | Organization | Individual (client, provider), Group/team (provider) | Quantitative and Qualitative | Individual (client), Group/team (provider), Organization | NR | Netherlands | National Funding Agency |
| Etz 2015^49^ | Randomized | Cluster Randomized Trial | Organization | Individual (provider), Organization | Quantitative and Qualitative | Individual (client, provider), Organization | Consolidated Framework for Implementation Research | USA | National Funding Agency |
| Ezeanolue 2013^50^ | Randomized | Cluster Randomized Trial | Organization | Individual (client) | Quantitative | Individual (client) | NR | Nigeria | National Funding Agency |
| Folks 2011^51^ | Randomized | Cluster Randomized Trial | Organization | Individual (client, provider) | Quantitative | Individual (client) | NR | USA | None |
| Fox 2013^52^ | Randomized | Cluster Randomized Trial | Organization | Organization | Quantitative and Qualitative | Individual (client), Organization | TRANSLATE framework | USA | National Funding Agency |
| Francis 2007^53^ | Randomized | Cluster Randomized Trial | Individual (provider) | Individual (provider) | Quantitative | Individual (provider) | NR | Canada | National Funding Agency |
| Fraser 2012^54^ | Quasi | Interrupted time series with controls | N/A | Individual (provider) | Quantitative and Qualitative | Individual (client), Organization | NR | Canada | National Funding Agency, Regional Funding Agency |
| Fulop 2013^55^ | Observational | Retrospective Case study | N/A | Organization | Quantitative and Qualitative | Individual (client), Organization | Greenhalgh Model of DOI in health organizations | England | National Funding Agency |
| Furler 2014^56^ | Randomized | Cluster Randomized Trial | Organization | Individual (provider), Organization | Quantitative | Individual (client) | Normalization Process Theory | Australia | National Funding Agency |
| Gagliardi 2014^57^ | Observational | Retrospective Case study | N/A | Organization | Quantitative and Qualitative | Individual (client), Group/Team (provider), Organization | Integrated/Interdisciplinary (unnamed) | Canada | Foundation |
| Gama 2015^58^ | Randomized | Randomized Controlled Trial | Individual (client) | Individual (provider) | Quantitative | Individual (client), Organization | NR | Malawi | Foundation |
| Garner 2010^59^ | Randomized | Cluster Randomized Trial | Organization | Individual (provider) | Quantitative | Individual (client, provider) | NR | USA | National Funding Agency |
| Gattelari 2011^60^ | Randomized | Cluster Randomized Trial | Organization | Individual (provider) | Quantitative | Organization | NR | Australia | National Funding Agency |
| Gattelari 2012^61^ | Randomized | Cluster Randomized Trial | Organization | Individual (provider) | Quantitative | Organization | NR | Australia | Foundation |
| Gifford 2008^62^ | Randomized | Cluster Randomized Trial | Group/Team (provider) | Organization | Quantitative and Qualitative | Individual (client, provider) | Ottawa Model of Research Use, Yukl effective leadership behaviors, and Gifford guideline implementation leadership | Canada | Regional Funding Agency, Foundation |
| Gold 2015^63^ | Randomized | Cluster Randomized Trial | Organization | Individual (provider), Organization | Quantitative and Qualitative | Individual (client, provider), Organization | RE-AIM | USA | National Funding Agency |
| Gong 2014^64^ | Randomized | Cluster Randomized Trial | Larger System Environment | Larger System Environment | Quantitative and Qualitative | Individual (client) | Self-developed case management model | China | Foundation |
| Green 2012^65^ | Randomized | Cluster Randomized Trial | Organization | Group/Team (provider) | Quantitative and Qualitative | Organization | Michie framework; Theoretical domains framework | Australia | Regional Agency |
| Gude 2017^66^ | Randomized | Cluster Randomized Trial | Group/team (provider) | Individual (provider), Group/team(provider) | Quantitative and Qualitative | Individual (client), Group/team (provider) | NR | Netherlands | Foundation |
| Hack 2011^67^ | Quasi | Pre-Post Without Controls | N/A | Individual (client) | Quantitative and Qualitative | Individual (client, provider) | PARIHS, Diffusion of Innovation, Social network theory | Canada | National Funding Agency |
| Hagedorn 2016^68^ | Quasi | Interrupted time series with controls | Organization | Individual (client, provider) | Quantitative and Qualitative | Individual (client, provider), Organization | Consolidated Framework for Implementation Research, | USA | National Funding Agency |
| Hall 2016^69^ | Randomized | Stepped Wedge Cluster Randomized Controlled Trial | Group/team (provider) | Individual (provider), Group/team (provider) | Quantitative and Qualitative | Individual (client, provider), Organization | i-PARIHS | Australia | National Funding Agency, Industry |
| Hamilton 2014^70^ | Randomized | Randomized Controlled Trial | Individual (client) | Individual (client, provider), Organization | Quantitative and Qualitative | Individual (client, provider), Organization | NR | USA | National Funding Agency |
| Hanbury 2010^71^ | Quasi | Interrupted time series with no controls | N/A | Individual (provider) | Quantitative and Qualitative | Individual (provider) | Greenhalgh Model of DOI in health organizations | UK | National Funding Agency |
| Hanson 2014^72,73^ | Quasi | Pre-Post With Controls | Larger System Environment | Group/team (provider), Organization | Quantitative and Qualitative | Individual (client, provider), Organization | NR | Tanzania, Uganda | International Agency (EU) |
| Harris 2013^74^ | Randomized | Cluster Randomized Trial | Organization | Organization | Quantitative and Qualitative | Individual (client, provider) | Multiple Organizational Process Theories; Chronic Care Model | Australia | National Funding Agency |
| Hartley 2017^75^ | Randomized | Cluster Randomized Factorial Trial | Organization | Individual (provider) | Quantitative | Individual (client), Organization | NR | UK | National Funding Agency |
| Hartzler 2017^76^ | Randomized | Randomized Controlled Trial | Individual (client) | Individual (client, provider) | Quantitative and Qualitative | Individual (client, provider), Organization | EPIS | USA | National Funding Agency |
| Hayek 2016^77^ | Randomized | Randomized Controlled Trial | Organization | Individual (provider) | Quantitative and Qualitative | Individual (client, provider) | NR | Australia | National Funding Agency |
| Herschell 2015^78^ | Randomized | Cluster Randomized Trial | Larger System Environment | Individual (provider), Organization | Quantitative and Qualitative | Individual (client, provider), Organization | Draft model of implementation research | USA | National Funding Agency |
| Heselmans 2013^79^ | Randomized | Cluster Randomized Trial | Organization | Individual (provider) | Quantitative | Individual (client) | NR | Belgium | National Funding Agency |
| Highfield 2015^80^ | Quasi | Nonrandomized Stepped Wedge Trial | Organization | Individual (provider), Organization | Quantitative | Individual (client), Organization | Consolidated Framework for Implementation Research | USA | National Funding Agency |
| Hocking 2016^81^ | Randomized | Cluster Randomized Factorial Trial | Organization | Individual (provider), Organization | Quantitative and Qualitative | Individual (client, provider), Organization | NR | Australia | National Funding Agency |
| Houston 2010^82^ | Randomized | Cluster Randomized Trial | Individual (client), Organization | Individual (client), Organization | Quantitative | Individual (client), Organization | Project Specific Conceptual Model | USA | National Funding Agency |
| Huis 2011^83^ | Randomized | Cluster Randomized Trial | Group/Team (provider) | Group/Team (provider) | Quantitative | Organization | NR | Netherlands | National Funding Agency |
| Hunter 2014^84^ | Observational | Cohort | N/A | Individual (provider) | Quantitative and Qualitative | Individual (client, provider), Organization, Larger System Environment | Consolidated Framework for Implementation Research | USA | National Funding Agency |
| Husebo 2015^85^ | Randomized | Cluster Randomized Trial | Organization | Individual (provider), Organization | Quantitative and Qualitative | Individual (client, provider) | NR | Norway | National Funding Agency, Foundation |
| Hutchinson 2015^86^ | Quasi | Interrupted time series with controls | Group/team (provider) | Individual (provider) | Quantitative and Qualitative | Individual (provider), Organization | PARIHS | Australia | National Funding Agency |
| Imms 2015^87^ | Quasi | Pre-Post With Controls | Organization | Individual (provider), Organization | Quantitative and Qualitative | Individual (client, provider), Organization | Diffusion, dissemination and implementation of innovation conceptual framework | Australia | National Funding Agency |
| Ista 2014^88^ | Quasi | Pre-Post Without Controls | N/A | Individual (client, provider) | Quantitative and Qualitative | Individual (client, provider) | Implementation frameworks by Grol and Wensing, and Cabana | Netherlands | National Funding Agency |
| Ivers 2010^89^ | Randomized | Cluster Randomized Trial | Organization | Individual (provider) | Quantitative and Qualitative | Individual (client) | Goal Setting Theory | Canada | National Funding Agency |
| Ivers 2012^90^ | Randomized | Cluster Randomized Trial | Individual (client) | Individual (client, provider) | Quantitative and Qualitative | Individual (client, provider) | NR | Canada | National Funding Agency |
| Ivers 2017^91^ | Randomized | Cluster Randomized Factorial Trial | Organization | Individual (provider) | Quantitative | Individual (client) | NR | Canada | National Funding Agency |
| Jabbour 2013^92^ | Randomized | Cluster Randomized Trial | Organization | Organization | Quantitative and Qualitative | Individual (client, provider) | Theoretical Domains Framework | Canada | National Funding Agency |
| Jabbour 2016^93^ | Quasi | Interrupted time series with no controls | N/A | Individual (provider), Organization | Quantitative and Qualitative | Individual (client), Group/team (provider), Organization | Theoretical Domains Framework | Canada | National Funding Agency |
| James 2013^94^ | Randomized | Cluster Randomized Trial | Organization | Individual (provider), Organization | Quantitative and Qualitative | Individual (client, provider), Organization | RE-AIM Framework | USA | National Funding Agency |
| Jensen 2014^95^ | Randomized | Cluster Randomized Trial | Organization | Individual (provider) | Quantitative | Individual (client), Organization | NR | Denmark | Foundation |
| Jeon 2013^96^ | Randomized | Cluster Randomized Trial | Organization | Group/Team (provider) | Quantitative | Group/Team (provider), Organization | ACLQF | Australia | National Funding Agency |
| Johnson 2006^97^ | Randomized | Cluster Randomized Trial | Organization | Individual (provider), Organization | Quantitative | Individual (client), Organization | NR | Canada | none |
| Jsbrandy 2015^98^ | Randomized | Cluster Randomized Trial | Organization | Individual (client, provider), Organization | Quantitative and Qualitative | Individual (client, provider), Organization | Grol and Wensing implementation of change model | Netherlands | Foundation |
| Kennedy 2012^99^ | Randomized | Cluster Randomized Trial | Organization | Individual (provider), Organization | Quantitative and Qualitative | Individual (client), Organization | Diffusion of Innovations | Canada | National Funding Agency |
| Keriel-Gascou 2013^100^ | Randomized | Stepped Wedge Cluster Randomized Controlled Trial | Individual (provider) | Organization | Quantitative | Individual (client, provider) | NR | France | none |
| Keurhorst 2013^101^ | Randomized | Cluster Randomized Factorial Trial | Organization | Organization | Quantitative | Individual (provider), Organization | NR | Netherlands, Sweden, Poland, England, Catalan | National Funding Agency |
| Kilbourne 2014^102^ | Randomized | Cluster Randomized SMART Implementation Trial | Organization | Individual (client, provider), Organization | Quantitative and Qualitative | Individual (client, provider), Organization | Rogers’ Diffusion of Innovations | USA | National Funding Agency |
| Knight 2016^103^ | Randomized | Cluster Randomized Trial | Organization | Individual (client, provider), Organization | Quantitative and Qualitative | Individual (client, provider), Organization | EPIS | USA | National Funding Agency |
| Krist 2014^104^ | Randomized | Cluster Randomized Trial | Organization | Individual (client, provider), Group/team (provider), Organization | Quantitative and Qualitative | Individual (client, provider), Group/team (provider), Organization | RE-AIM | USA | National Funding Agency |
| Lavis 2011^105^ | Randomized | Randomized Controlled Trial | Individual (provider) | Individual (provider), Group/Team (provider) | Quantitative and Qualitative | Individual (provider), Group/Team (provider) | NR | Canada | Regional Funding Agency |
| Lee 2016^106^ | Randomized | Cluster Randomized Trial | Organization | Individual (provider) | Quantitative and Qualitative | Individual (client, provider) | Normalization Process Theory | Canada | Foundation |
| Lewis 2015^107^ | Randomized | Dynamic Randomized Controlled Trial | Organization | Individual (client, provider), Organization | Quantitative and Qualitative | Individual (client, provider), Organization | Framework for Dissemination, Dynamic Sustainability Framework | USA | National Funding Agency |
| Li 2014^108^ | Quasi | Pre-Post Without Controls | N/A | Individual (client, provider) | Quantitative | Individual (client, provider), Organization | RE-AIM | USA | National Funding Agency |
| Liddy 2011^109^ | Randomized | Stepped Wedge Cluster Randomized Controlled Trial | Organization | Organization | Quantitative and Qualitative | Individual (client) | Chronic Care Model | Canada | Regional Funding Agency |
| Liu 2013^110^ | Quasi | Interrupted time series with no controls | N/A | Organization | Quantitative and Qualitative | Individual (client) | Knowledge to Action Framework | Canada | none |
| Lubinga 2014^111^ | Quasi | Pre-Post With Controls | Larger System Environment | Individual (provider), Organization | Quantitative | Individual (client), Organization | NR | Malawi | Foundation |
| Luitjes 2010^112^ | Randomized | Cluster Randomized Trial | Organization | Individual (provider) | Quantitative and Qualitative | Individual (client) | NR | Netherlands | National Funding Agency |
| MacDermid 2006^113^ | Quasi | Phased Implementation | Individual (provider) | Individual (provider) | Quantitative and Qualitative | Individual (provider) | NR | Canada | National Funding Agency |
| Macdermind 2012^114^ | Randomized | Randomized Crossover | Individual (provider) | Individual (provider) | Quantitative and Qualitative | Individual (provider) | Conceptual Framework of Technology-Enabled KT | Canada | National Funding Agency |
| Mann 2011^115^ | Randomized | Randomized Controlled Trial | Individual (provider) | Individual (provider) | Quantitative | Individual (client, provider) | NR | USA | National Funding Agency |
| Mann 2012^116^ | Randomized | Randomized Controlled Trial | Individual (provider) | Individual (client, provider) | Quantitative | Individual (client, provider) | NR | USA | National Funding Agency |
| Martino 2015^117^ | Randomized | Randomized Controlled Trial | Individual (provider) | Individual (client, provider) | Quantitative and Qualitative | Individual (client, provider) | NR | USA | National Funding Agency |
| McAlister 2006^118^ | Randomized | Cluster Randomized Trial | Individual (client), Organization | Individual (provider) | Quantitative | Individual (client, provider) | NR | Canada | National Funding Agency, Regional Funding Agency |
| McEwen 2015 - Study 1^119^ | Quasi | Interrupted time series with no controls | Individual (client) | Individual (client, provider), Organization | Quantitative | Individual (client) | Knowledge to Action Framework | Canada | NR |
| McEwen 2015 - Study 2^119^ | Quasi | Pre-Post Without Controls | Individual (provider) | Individual (client, provider), Organization | Quantitative and Qualitative | Individual (provider), Group/team (provider) | Knowledge to Action Framework | Canada | NR |
| McEwen 2015 - Study 3^119^ | Quasi | Pre-Post With Controls | Individual (client) | Individual (client, provider), Organization | Quantitative | Individual (client) | Knowledge to Action Framework | Canada | NR |
| McKenzie 2008^120^ | Randomized | Cluster Randomized Trial | Organization | Individual (provider) | Quantitative | Individual (provider), Organization | NR | Australia | National Funding Agency |
| Mckenzie 2010^121^ | Randomized | Cluster Randomized Trial | Organization | Individual (provider) | Quantitative | Individual (client, provider) | NR | Australia | National Funding Agency |
| McKenzie 2013^122^ | Randomized | Cluster Randomized Trial | Organization | Individual (provider) | Quantitative | Individual (client, provider) | NR | Australia | National Funding Agency |
| Melman 2013^123^ | Quasi | Pre-Post With Controls | Organization | Individual (provider) | Quantitative and Qualitative | NR | NR | Netherlands | National Funding Agency |
| Menya 2013^124^ | Randomized | Cluster Randomized Trial | Organization | Organization | Quantitative | Organization | NR | Kenya | National Funding Agency |
| Mettes 2007^125^ | Randomized | Cluster Randomized Trial | Group/Team (provider) | Group/Team (provider) | Quantitative | Individual (provider) | NR | Netherlands | National Agency |
| Middleton 2009^126^ | Randomized | Cluster Randomized Trial | Organization | Organization | Quantitative | Individual (client, provider) | Knowledge Translation Theory | Australia | National Funding Agency |
| Middleton 2016^127^ | Randomized | Cluster Randomized Trial | Group/team (provider) | Individual (provider), Group/team (provider) | Quantitative and Qualitative | Individual (client), Group/team (provider) | Theoretical Domains Framework | Australia | National Funding Agency |
| Minian 2017^128^ | Randomized | Cluster Randomized Trial | Organization | Individual (client, provider) | Quantitative and Qualitative | Individual (client, provider) | NR | Canada | Foundation |
| Moja 2008^129^ | Randomized | Randomized Controlled Trial | Individual (provider) | Individual (provider) | Quantitative | Individual (provider) | Modified version of Miller's Model | Italy | National Agency |
| Moja 2016 - CODES^130^ | Randomized | Randomized Controlled Trial | Individual (client) | Individual (provider) | Quantitative | Individual (client) | NR | Italy | National Funding Agency |
| Moja 2016 - ONCO-CODES^131^ | Randomized | Randomized Controlled Trial | Individual (client) | Individual (provider) | Quantitative | Individual (client) | NR | Italy | National Funding Agency |
| Molfenter 2013^132^ | Randomized | Cluster Randomized Trial | Organization | Organization | Quantitative | Organization | Advancing Recovery Framework | USA | National Funding Agency |
| Mortimer 2008^133^ | Randomized | Cluster Randomized Trial | Organization | Organization | Quantitative | Individual (client) | NR | Australia | National Funding Agency |
| Nouwens 2012^134^ | Randomized | Cluster Randomized Trial | Organization | Individual (provider) | Quantitative and Qualitative | Individual (client, provider) | NR | Netherlands | National Funding Agency |
| Ober 2015^135^ | Randomized | Randomized Controlled Trial | Individual (provider) | Individual (provider), Organization | Quantitative and Qualitative | Individual (client, provider) | Chronic Care Model | USA | National Funding Agency |
| Osteras 2015^136^ | Randomized | Stepped Wedge Cluster Randomized Controlled Trial | Larger System Environment | Individual (client, provider) | Quantitative and Qualitative | Individual (client, provider) | NR | Norway | National Funding Agency |
| Ostroff 2014^137^ | Randomized | Cluster Randomized Trial | Organization | Organization | Quantitative and Qualitative | Organization | Organizational Change Model, Theory of Planned Behavior | USA | National Funding Agency |
| Owen 2013^138^ | Randomized | Cluster Randomized Trial | Organization | Organization | Quantitative | Organization | PARIHS Framework | USA | National Agency |
| Parchman 2008^139^ | Randomized | Cluster Randomized Trial | Organization | Organization | Quantitative | Organization | Complex Adaptive Systems (CAS) Theory | USA | National Funding Agency |
| Parchman 2016^140^ | Randomized | 2X2 Randomized Factorial Design | Organization | Individual (client, provider), Group/team (provider), Organization | Quantitative and Qualitative | Individual (client, provider), Organization | Diffusion of innovation | USA | National Funding Agency |
| Paul 2013^141^ | Randomized | Cluster Randomized Trial | Larger System Environment | Individual (provider) | Quantitative | Larger System Environment | NR | Australia | National Funding Agency |
| Paul 2014^142^ | Randomized | Cluster Randomized Trial | Organization | Individual (provider), Organization | Quantitative | Organization | NR | Australia | National Funding Agency, Industry |
| Peiris 2016^143^ | Randomized | Cluster Randomized Trial | Larger System Environment | Individual (client, provider) | Quantitative and Qualitative | Individual (client), Organization | Normalisation Process Theory | China | National Funding Agency |
| Pellecchia 2016^144^ | Randomized | Cluster Randomized Trial | Individual (provider) | Individual (client, provider) | Quantitative and Qualitative | Individual (client, provider) | NR | USA | National Funding Agency |
| Petersen 2011^145^ | Randomized | Randomized Controlled Trial | Organization | Individual (provider), Organization | Quantitative | Individual (provider) | NR | USA | National Funding Agency, National Agency |
| Pinto 2014^146^ | Randomized | Cluster Randomized Trial | Organization | Individual (provider) | Quantitative | Individual (client, provider) | NR | Portugal | National Funding Agency |
| Prados-Torres 2017^147^ | Randomized | Cluster Randomized Trial | Individual (provider) | Individual (provider) | Quantitative | Individual (client) | NR | Spain | National Funding Agency, Regional Funding Agency |
| Praveen 2013^148^ | Randomized | Cluster Randomized Trial | Organization | Individual (provider), Organization | Quantitative | Individual (client, provider) | NR | India | National Funding Agency, International Funding Agency |
| Prior 2014^149^ | Randomized | Partial factorial cluster randomised trial | Organization | Individual (provider) | Quantitative and Qualitative | Individual (provider), Organization | Consolidated Framework for Implementation Research, Theoretical Domains Framework | UK | National Funding Agency |
| Pugh 2014^150^ | Observational | Comparative case study | N/A | Individual (provider), Organization | Quantitative and Qualitative | Individual (client, provider), Group/team (provider), Organization | NR | USA | National Funding Agency |
| Quanbeck 2011^151^ | Randomized | Cluster Randomized Trial | Organization | Individual (provider) | Quantitative and Qualitative | Organization | NR | USA | National Funding Agency |
| Quanbeck 2014^152^ | Quasi | Multiple Baseline Design | Organization | Individual (client, provider), Organization | Quantitative and Qualitative | Individual (client, provider), Organization | Diffusion of innovations, RE-AIM | USA | National Funding Agency |
| Rabbani 2014^153^ | Randomized | Cluster Randomized Trial | Individual (provider) | Individual (provider) | Quantitative and Qualitative | Individual (provider) | NR | Pakistan | International Agency |
| Ramsey 2015^154^ | Quasi | Interrupted time series with no controls | Group/team (provider) | Individual (client, provider) | Quantitative and Qualitative | Individual (client, provider) | PARIHS | USA | National Funding Agency |
| Ranta 2012^155^ | Randomized | Cluster Randomized Trial | Organization | Individual (provider) | Quantitative | Individual (client, provider) | NR | New Zealand | National Funding Agency |
| Ridde 2014^156^ | Observational | Multiple case study | N/A | Individual (client, provider) | Quantitative and Qualitative | Individual (client, provider), Organization, Larger System Environment | NR | Burkina Faso | National Funding Agency |
| Rijis 2013^157^ | Randomized | Cluster Randomized Trial | Organization | Organization | Quantitative | Individual (client, provider) | NR | Denmark | Foundation |
| Rosella 2014^158^ | Observational | Multiple case study | N/A | Individual (provider), Group/team (provider) | Quantitative and Qualitative | Individual (provider), Group/team (provider) | Knowledge to Action Framework | Canada | National Funding Agency |
| Sales 2016^159^ | Quasi | Interrupted time series with controls | Organization | Individual (provider), Group/team (provider) | Quantitative and Qualitative | Individual (client, provider), Organization | Consolidated Framework for Implementation Research, Theoretical Domains Framework | USA | National Funding Agency |
| Scales 2009^160^ | Randomized | Cluster Randomized Trial | Organization | Organization | Quantitative | Organization | NR | Canada | Regional Funding Agency |
| Scott 2009-Evaluation^161^ | Randomized | Cluster Randomized Trial | Organization | Individual (provider) | Quantitative | Individual (client, provider) | NR | Canada | National Funding Agency |
| Scott 2009-Management^161^ | Randomized | Cluster Randomized Trial | NR | NR | Quantitative and Qualitative | Individual (provider), Organization | NR | Canada | National Funding Agency |
| Scott 2009-Storytelling^161^ | Randomized | Randomized Controlled Trial | Organization | Individual (client) | Quantitative | Individual (client) | NR | Canada | National Funding Agency |
| Seers 2012^162^ | Randomized | Randomized Controlled Trial | Organization | Group/Team (provider) | Quantitative and Qualitative | Individual (client), Group/Team (provider), Organization | Promoting Action on Research Implementation in PARIHS Framework | UK, Sweden, Netherlands, and Republic of Ireland | International Agency |
| Shelley 2015^163^ | Randomized | Cluster Randomized Trial | Organization | Individual (client, provider) | Quantitative and Qualitative | Individual (client, provider), Organization | Consolidated Framework for Implementation Research | Vietnam | National Funding Agency |
| Shelley 2016^164^ | Randomized | Stepped Wedge Cluster Randomized Controlled Trial | Organization | Individual (provider), Group/team (provider) | Quantitative and Qualitative | Individual (client, provider), Organization, Larger System Environment | Consolidated Framework for Implementation Research | USA | National Funding Agency |
| Sherr 2014^165^ | Randomized | Cluster Randomized Trial | Organization | Organization | Quantitative and Qualitative | Individual (provider), Organization | Consolidated Framework for Implementation Research | Côte d’Ivoire, Kenya and Mozambique | National Funding Agency, Foundation |
| Siebenhofer 2012^166^ | Randomized | Cluster Randomized Trial | Organization | Individual (client), Group/Team (provider) | Quantitative | Individual (client, provider), Group/Team (provider) | NR | Germany | National Funding Agency |
| Simmons 2017^167^ | Randomized | Cluster Randomized Trial | Organization | Individual (client, provider), Organization | Quantitative and Qualitative | Individual (client, provider), Organization | Consolidated Framework for Implementation Research | USA | National Funding Agency |
| Sinnema 2011^168^ | Randomized | Cluster Randomized Trial | Organization | Individual (provider) | Quantitative and Qualitative | Organization | NR | Netherlands | National Funding Agency |
| Smelson 2015^169^ | Randomized | Cluster Randomized Trial | Group/team (provider) | Individual (client, provider), Group/team (provider) | Quantitative | Individual (client, provider) | Consolidated Framework for Implementation Research, RE-AIM | USA | National Funding Agency |
| Smith 2013^170^ | Randomized | Cluster Randomized Trial | Organization | Individual (client, provider), Organization | Quantitative | Larger System Environment | Rogers’ DOI and RE-AIM | USA | National Funding Agency |
| Spaulding 2009^171^ | Quasi | Interrupted time series with controls | Organization | Individual (client, provider) | Quantitative | Individual (client, provider) | NR | USA | National Funding Agency, Internal |
| Squires 2013^172^ | Quasi | Pre-Post Without Controls | Organization | NR | Quantitative and Qualitative | Individual (client) | UK MRC Complex Interventions Framework | Canada | Foundation |
| Squires 2014^173^ | Quasi | Interrupted time series with no controls | Larger System Environment | Organization | Quantitative | Organization, Larger System Environment | Theoretical Domains Framework, Knowledge to action | Canada | National Funding Agency |
| Stacey 2012^174^ | Observational | Comparative case study | N/A | Group/Team (provider) | Quantitative and Qualitative | Individual (client), Group/Team (provider), Organization | Knowledge to Action Framework | Canada | National Funding Agency |
| Stacey 2016^175^ | Observational | Comparative case study | N/A | Individual (client, provider) | Quantitative and Qualitative | Individual (client, provider) | NR | Canada | National Funding Agency |
| Staedke 2013^176^ | Randomized | Cluster Randomized Trial | Organization | Individual (provider), Group/Team (provider) | Quantitative | Organization | NR | Uganda | Foundation |
| Stiell 2007^177^ | Randomized | Cluster Randomized Trial | Organization | Individual (provider) | Quantitative | Individual (client, provider), Organization | NR | Canada | National Funding Agency |
| Stienen 2013^178^ | Randomized | Cluster Randomized Trial | Organization | Individual (client, provider) | Quantitative and Qualitative | Individual (client, provider) | Framework developed by Cabana and Grol [Cabana, 1999; Grol, 2004] | Netherlands | National Funding Agency |
| Stirman 2013^179^ | Randomized | Randomized Controlled Trial | Individual (provider) | Individual (provider) | Quantitative and Qualitative | Individual (client, provider) | Consolidated Framework for Implementation Research | Canada | National Funding Agency |
| Swindle 2017^180^ | Randomized | Cluster Randomized Trial | Organization | Individual (provider) | Quantitative and Qualitative | Individual (client, provider) | i-PARIHS, RE-AIM | USA | National Funding Agency |
| Tapp 2014^181^ | Randomized | Cluster Randomized Trial | Organization | Individual (client, provider) | Quantitative and Qualitative | Individual (client, provider) | NR | USA | National Funding Agency |
| te Boveldt 2011^182^ | Randomized | Cluster Randomized Trial | Organization | Individual (client, provider) | Quantitative and Qualitative | Individual (client, provider), Organization | NR | Netherlands | National Funding Agency |
| Tello-Bernabe 2011^183^ | Randomized | Cluster Randomized Trial | Organization | Group/Team (provider) | Quantitative and Qualitative | Individual (client), Group/Team (provider), Organization | NR | Spain | National Funding Agency |
| Toftegaard 2014^184^ | Randomized | Stepped Wedge Cluster Randomized Controlled Trial | Larger System Environment | Individual (provider) | Quantitative | Individual (client, provider) | NR | Denmark | Regional Funding Agency, Foundation |
| Trautner 2011^185^ | Quasi | Pre-Post With Controls | Organization | Individual (provider), Organization | Quantitative and Qualitative | Individual (provider), Organization | Cabana et al. [Cabana, 1999,] Model of barriers to physician guideline adherence | USA | National Funding Agency, National Agency, Foundation |
| Tremblay 2010^186^ | Quasi | Pre-Post Without Controls | N/A | N/A | Quantitative and Qualitative | Individual (client), Group/Team (client, provider) | Ottawa Model of Research Use | Canada | Foundation |
| Treweek 2011^187^ | Randomized | Randomized Controlled Trial | Individual (provider) | Individual (provider) | Quantitative and Qualitative | Individual (client) | NR | Scotland | National Funding Agency |
| Trietsch 2009^188^ | Randomized | Cluster Randomized Trial | Organization | Individual (provider), Group/Team (provider) | Quantitative | Individual (provider), Group/Team (provider) | Grol R, 2005 | Netherlands | National Funding Agency |
| Van de Steeg 2012^189^ | Randomized | Stepped Wedge Cluster Randomized Controlled Trial | Organization | Group/Team (provider) | Quantitative | Individual (provider), Organization | NR | Netherlands | National Agency |
| van den Boogaard 2013^190^ | Randomized | Cluster Randomized Trial | Organization | Individual (client, provider), Organization | Quantitative | Individual (client), Organization | NR | Netherlands | National Funding Agency |
| van Engen-Verheul 2014^191^ | Randomized | Stepped Wedge Cluster Randomized Controlled Trial | Organization | Individual (provider), Organization | Quantitative and Qualitative | Individual (client), Organization | NR | Netherlands | National Funding Agency |
| van Gaalen 2012^192^ | Randomized | Cluster Randomized Trial | Organization | Organization | Quantitative | Individual (client), Organization | NR | Netherlands | National Funding Agency, Foundation |
| van Lieshout 2011^193^ | Randomized | Cluster Randomized Trial | Individual (provider) | Individual (provider) | Quantitative and Qualitative | Individual (client, provider) | Conceptual framework with knowledge, attitude, and behaviour (Légaré, 2008) | Netherlands | National Agency |
| Voorn 2014^194^ | Randomized | Stepped Wedge Cluster Randomized Controlled Trial | Organization | Individual (provider) | Quantitative | Individual (client, provider) | Theoretical Domains Framework | Netherlands | National Funding Agency |
| Waldorff 2011^195^ | Randomized | Randomized Controlled Trial | Individual (provider), Organization | Organization | Quantitative | Organization | NR | Denmark | Regional Agency |
| Watson 2014^196^ | Quasi | Pre-Post Without Controls | Organization | Individual (provider), Organization | Quantitative and Qualitative | Individual (provider), Organization, Larger System Environment | Proctor's Conceptual model of implementation research | USA | National Funding Agency |
| Weiner 2015^197^ | Randomized | Stepped Wedge Cluster Randomized Controlled Trial | Organization | Individual (provider), Organization | Quantitative and Qualitative | Individual (client, provider), Organization | NR | USA | National Funding Agency |
| Wensing 2009^198^ | Observational | Cross-Sectional | Group/Team (provider) | N/A | Quantitative | Individual (client, provider), Group/Team (provider), Organization | NR | Austria, Belgium, England, Finland, France, Germany, Netherlands, Slovenia, Spain, and Switzerland | Foundation |
| Willging 2016^199^ | Randomized | Cluster Randomized Trial | Organization | Individual (client, provider), Organization | Quantitative and Qualitative | Individual (client, provider), Organization, Larger System Environment | EPIS | USA | National Funding Agency |
| Williams 2013^200^ | Randomized | Cluster Randomized Trial | Organization | Group/Team (provider) | Quantitative and Qualitative | Group/Team (provider) | SWOT Analysis’ developed by Albert Humphrey | Australia | National Funding Agency |
| Willis 2016^201^ | Randomized | Cluster Randomized Trial | Organization | Individual (provider), Organization | Quantitative and Qualitative | Individual (client, provider), Organization | Theoretical Domains Framework | UK | National Funding Agency |
| Wilson 2011^202^ | Randomized | Randomized Controlled Trial | Organization | Group/Team (provider) | Quantitative and Qualitative | Organization | NR | Canada | none |
| Wilson 2015^203^ | Quasi | Pre-Post With Controls | Organization | Individual (provider), Organization | Quantitative and Qualitative | Individual (provider), Organization | NR | UK | National Funding Agency |
| Wiltsey Stirman 2017^204^ | Randomized | Cluster Randomized Trial | Organization | Individual (provider), Organization | Quantitative and Qualitative | Individual (client, provider), Organization | Dynamic Sustainability Framework | USA, Canada | National Funding Agency |
| Wolfenden 2014^205^ | Randomized | Cluster Randomized Trial | Organization | Organization | Quantitative | Organization | Theoretical Domains Framework | Australia | National Funding Agency |
| Wu 2015^206^ | Quasi | Pre-Post Without Controls | Organization | Individual (client, provider), Organization | Quantitative and Qualitative | Individual (client, provider), Organization | RE-AIM, Weiner organizational model of innovation implementation | USA | National Funding Agency |
| Yano 2016^207^ | Randomized | Cluster Randomized Trial | Organization | Individual (provider), Group/team (provider), Organization | Quantitative and Qualitative | Individual (client, provider), Organization | Diffusion of Innovation | USA | National Funding Agency |
| Zatzick 2016^208^ | Randomized | Stepped Wedge Cluster Randomized Controlled Trial | Organization | Individual (provider), Organization | Quantitative and Qualitative | Individual (client, provider), Organization | RE-AIM, Diffusion of innovation | USA | National Funding Agency |
| Zwarenstein 2007^209^ | Randomized | Cluster Randomized Trial | Organization | Individual (provider) | Quantitative | Individual (client, provider) | NR | Canada | National Funding Agency |

**References**

1. Aarons GA, Green AE, Palinkas LA, et al. Dynamic adaptation process to implement an evidence-based child maltreatment intervention. *Implementation Science.* 2012;7(1):32.

2. Aarons GA, Ehrhart MG, Moullin JC, Torres EM, Green AE. Testing the leadership and organizational change for implementation (LOCI) intervention in substance abuse treatment: a cluster randomized trial study protocol. *Implementation science : IS.* 2017;12(1):29.

3. Acolet D, Jelphs K, Davidson D, et al. The BLISS cluster randomised controlled trial of the effect of 'active dissemination of information' on standards of care for premature babies in England (BEADI) study protocol [ISRCTN89683698]. *Implementation science : IS.* 2007;2:33.

4. Agoritsas T, Iserman E, Hobson N, et al. Increasing the quantity and quality of searching for current best evidence to answer clinical questions: protocol and intervention design of the MacPLUS FS Factorial Randomized Controlled Trials. *Implementation science : IS.* 2014;9:125.

5. Allen P, Sequeira S, Jacob RR, et al. Promoting state health department evidence-based cancer and chronic disease prevention: a multi-phase dissemination study with a cluster randomized trial component. *Implementation science : IS.* 2013;8:141.

6. Amemori M, Korhonen T, Kinnunen T, Michie S, Murtomaa H. Enhancing implementation of tobacco use prevention and cessation counselling guideline among dental providers: a cluster randomised controlled trial. *Implementation science : IS.* 2011;6:13.

7. Anderson RA, Corazzini K, Porter K, Daily K, McDaniel RR, Jr., Colon-Emeric C. CONNECT for quality: protocol of a cluster randomized controlled trial to improve fall prevention in nursing homes. *Implementation science : IS.* 2012;7:11.

8. Anrys P, Strauven G, Boland B, et al. Collaborative approach to Optimise MEdication use for Older people in Nursing homes (COME-ON): study protocol of a cluster controlled trial. *Implementation science : IS.* 2016;11:35.

9. Aron DC, Lowery J, Tseng CL, Conlin P, Kahwati L. De-implementation of inappropriately tight control (of hypoglycemia) for health: protocol with an example of a research grant application. *Implementation science : IS.* 2014;9:58.

10. Bartlem K, Bowman J, Freund M, et al. Evaluating the effectiveness of a clinical practice change intervention in increasing clinician provision of preventive care in a network of community-based mental health services: a study protocol of a non-randomized, multiple baseline trial. *Implementation science : IS.* 2013;8:85.

11. Bauer MS, Miller C, Kim B, et al. Partnering with health system operations leadership to develop a controlled implementation trial. *Implementation science : IS.* 2016;11:22.

12. Beidas RS, Aarons G, Barg F, et al. Policy to implementation: evidence-based practice in community mental health--study protocol. *Implementation science : IS.* 2013;8:38.

13. Blanchet K, Lewis JJ, Pozo-Martin F, et al. A Quantitative and Qualitative methods protocol to evaluate the effect and cost-effectiveness of an Integrated electronic Diagnosis Approach (IeDA) for the management of childhood illnesses at primary health facilities in Burkina Faso. *Implementation science : IS.* 2016;11(1):111.

14. Boivin A, Lehoux P, Lacombe R, Lacasse A, Burgers J, Grol R. Target for improvement: a cluster randomised trial of public involvement in quality-indicator prioritisation (intervention development and study protocol). *Implementation science : IS.* 2011;6:45.

15. Borgermans L, Goderis G, Van Den Broeke C, et al. A cluster randomized trial to improve adherence to evidence-based guidelines on diabetes and reduce clinical inertia in primary care physicians in Belgium: study protocol [NTR 1369]. *Implementation science : IS.* 2008;3:42.

16. Borghi J, Mayumana I, Mashasi I, et al. Protocol for the evaluation of a pay for performance programme in Pwani region in Tanzania: a controlled before and after study. *Implementation science : IS.* 2013;8(80):5908-5908.

17. Botti M, Kent B, Bucknall T, et al. Development of a Management Algorithm for Post-operative Pain (MAPP) after total knee and total hip replacement: study rationale and design. *Implementation science : IS.* 2014;9:110.

18. Bower P, Kennedy A, Reeves D, et al. A cluster randomised controlled trial of the clinical and cost-effectiveness of a 'whole systems' model of self-management support for the management of long- term conditions in primary care: trial protocol. *Implementation science : IS.* 2012;7:7.

19. Brouwers MC, Makarski J, Levinson AJ. A randomized trial to evaluate e-learning interventions designed to improve learner's performance, satisfaction, and self-efficacy with the AGREE II. *Implementation science : IS.* 2010;5:29.

20. Brown BB, Young J, Smith DP, et al. Clinician-led improvement in cancer care (CLICC)--testing a multifaceted implementation strategy to increase evidence-based prostate cancer care: phased randomised controlled trial--study protocol. *Implementation science : IS.* 2014;9:64.

21. Bucknall TK, Harvey G, Considine J, et al. Prioritising Responses Of Nurses To deteriorating patient Observations (PRONTO) protocol: testing the effectiveness of a facilitation intervention in a pragmatic, cluster-randomised trial with an embedded process evaluation and cost analysis. *Implementation science : IS.* 2017;12(1):85.

22. Bunker JM, Reddel HK, Dennis SM, et al. A pragmatic cluster randomized controlled trial of early intervention for chronic obstructive pulmonary disease by practice nurse-general practitioner teams: Study Protocol. *Implementation science : IS.* 2012;7:83.

23. Campbell-Scherer DL, Asselin J, Osunlana AM, et al. Implementation and evaluation of the 5As framework of obesity management in primary care: design of the 5As Team (5AsT) randomized control trial. *Implementation science : IS.* 2014;9:78.

24. Charrois TL, McAlister FA, Cooney D, et al. Improving hypertension management through pharmacist prescribing; the rural Alberta clinical trial in optimizing hypertension (Rural RxACTION): trial design and methods. *Implementation science : IS.* 2011;6:94.

25. Chinman M, Daniels K, Smith J, et al. Provision of peer specialist services in VA patient aligned care teams: protocol for testing a cluster randomized implementation trial. *Implementation science : IS.* 2017;12(1):57.

26. Clemson L, Mackenzie L, Roberts C, et al. Integrated solutions for sustainable fall prevention in primary care, the iSOLVE project: a type 2 hybrid effectiveness-implementation design. *Implementation science : IS.* 2017;12(1):12.

27. Cohen DJ, Balasubramanian BA, Gordon L, et al. A national evaluation of a dissemination and implementation initiative to enhance primary care practice capacity and improve cardiovascular disease care: the ESCALATES study protocol. *Implementation science : IS.* 2016;11(1):86.

28. Cooper LA, Roter DL, Bone LR, et al. A randomized controlled trial of interventions to enhance patient-physician partnership, patient adherence and high blood pressure control among ethnic minorities and poor persons: study protocol NCT00123045. *Implementation Science.* 2009;4(1):7.

29. Cooper LA, Marsteller JA, Noronha GJ, et al. A multi-level system quality improvement intervention to reduce racial disparities in hypertension care and control: study protocol. *Implementation science : IS.* 2013;8:60.

30. Couturier J, Kimber M, Lock J, et al. Implementing highly specialized and evidence-based pediatric eating disorder treatment: protocol for a Quantitative and Qualitative methods evaluation. *Implementation science : IS.* 2015;10:40.

31. Cowan JF, Micek M, Cowan JF, et al. Early ART initiation among HIV-positive pregnant women in central Mozambique: a stepped wedge randomized controlled trial of an optimized Option B+ approach. *Implementation science : IS.* 2015;10:61.

32. Cucciare MA, Curran GM, Craske MG, et al. Assessing fidelity of cognitive behavioral therapy in rural VA clinics: design of a randomized implementation effectiveness (hybrid type III) trial. *Implementation science : IS.* 2016;11:65.

33. Cully JA, Armento ME, Mott J, et al. Brief cognitive behavioral therapy in primary care: a hybrid type 2 patient-randomized effectiveness-implementation design. *Implementation science : IS.* 2012;7:64.

34. Dainty KN, Scales DC, Brooks SC, et al. A knowledge translation collaborative to improve the use of therapeutic hypothermia in post-cardiac arrest patients: protocol for a stepped wedge randomized trial. *Implementation science : IS.* 2011;6:4.

35. Damschroder LJ, Moin T, Datta SK, et al. Implementation and evaluation of the VA DPP clinical demonstration: protocol for a multi-site non-randomized hybrid effectiveness-implementation type III trial. *Implementation science : IS.* 2015;10:68.

36. de Groot JJ, Maessen JM, Slangen BF, Winkens B, Dirksen CD, van der Weijden T. A stepped strategy that aims at the nationwide implementation of the Enhanced Recovery After Surgery programme in major gynaecological surgery: study protocol of a cluster randomised controlled trial. *Implementation science : IS.* 2015;10:106.

37. de Lusignan S, Gallagher H, Chan T, et al. The QICKD study protocol: a cluster randomised trial to compare quality improvement interventions to lower systolic BP in chronic kidney disease (CKD) in primary care. *Implementation science : IS.* 2009;4:39.

38. Dekker N, Hermens RP, Elwyn G, et al. Improving calculation, interpretation and communication of familial colorectal cancer risk: protocol for a randomized controlled trial. *Implementation science : IS.* 2010;5:6.

39. Del Cura-Gonzalez I, Lopez-Rodriguez JA, Sanz-Cuesta T, et al. Effectiveness of a strategy that uses educational games to implement clinical practice guidelines among Spanish residents of family and community medicine (e-EDUCAGUIA project): a clinical trial by clusters. *Implementation science : IS.* 2016;11:71.

40. Deneckere S, Euwema M, Lodewijckx C, Panella M, Sermeus W, Vanhaecht K. The European quality of care pathways (EQCP) study on the impact of care pathways on interprofessional teamwork in an acute hospital setting: study protocol: for a cluster randomised controlled trial and evaluation of implementation processes. *Implementation science : IS.* 2012;7:47.

41. DeVoe JE, Huguet N, Likumahuwa-Ackman S, et al. Testing health information technology tools to facilitate health insurance support: a protocol for an effectiveness-implementation hybrid randomized trial. *Implementation science : IS.* 2015;10:123.

42. Dorr DA, McConnell KJ, Williams MP, et al. Study protocol: transforming outcomes for patients through medical home evaluation and redesign: a cluster randomized controlled trial to test high value elements for patient-centered medical homes versus quality improvement. *Implementation science : IS.* 2015;10:13.

43. Dorsey S, Pullmann MD, Deblinger E, et al. Improving practice in community-based settings: a randomized trial of supervision - study protocol. *Implementation science : IS.* 2013;8:89.

44. Eccles MP, Johnston M, Hrisos S, et al. Translating clinicians' beliefs into implementation interventions (TRACII): a protocol for an intervention modeling experiment to change clinicians' intentions to implement evidence-based practice. *Implementation science : IS.* 2007;2:27.

45. Eiraldi R, McCurdy B, Khanna M, et al. A cluster randomized trial to evaluate external support for the implementation of positive behavioral interventions and supports by school personnel. *Implementation science : IS.* 2014;9:12.

46. Eiraldi R, Khanna MS, Jawad AF, et al. A hybrid effectiveness-implementation cluster randomized trial of group CBT for anxiety in urban schools: rationale, design, and methods. *Implementation science : IS.* 2016;11:92.

47. Elliot DL, Kerry KS, Moe EL, et al. The IGNITE (investigation to guide new insight into translational effectiveness) trial: Protocol for a translational study of an evidenced-based wellness program in fire departments. *Implementation science : IS.* 2010;5:73.

48. Emond YE, Calsbeek H, Teerenstra S, et al. Improving the implementation of perioperative safety guidelines using a multifaceted intervention approach: protocol of the IMPROVE study, a stepped wedge cluster randomized trial. *Implementation science : IS.* 2015;10:3.

49. Etz RS, Keith RE, Maternick AM, et al. Supporting Practices to Adopt Registry-Based Care (SPARC): protocol for a randomized controlled trial. *Implementation science : IS.* 2015;10:46.

50. Ezeanolue EE, Obiefune MC, Yang W, Obaro SK, Ezeanolue CO, Ogedegbe GG. Comparative effectiveness of congregation- versus clinic-based approach to prevention of mother-to-child HIV transmission: study protocol for a cluster randomized controlled trial. *Implementation science : IS.* 2013;8:62.

51. Folks B, Leblanc WG, Staton EW, Pace WD. Reconsidering low-dose aspirin therapy for cardiovascular disease: a study protocol for physician and patient behavioral change. *Implementation science : IS.* 2011;6:65.

52. Fox CH, Vest BM, Kahn LS, et al. Improving evidence-based primary care for chronic kidney disease: study protocol for a cluster randomized control trial for translating evidence into practice (TRANSLATE CKD). *Implementation science : IS.* 2013;8:88.

53. Francis JJ, Grimshaw JM, Zwarenstein M, et al. Testing a TheoRY-inspired MEssage ('TRY-ME'): a sub-trial within the Ontario Printed Educational Message (OPEM) trial. *Implementation science : IS.* 2007;2:39.

54. Fraser KD, Sales AE, O'Rourke HM, Schalm C. Data for improvement and clinical excellence: protocol for an audit with feedback intervention in home care and supportive living. *Implementation science : IS.* 2012;7:4.

55. Fulop N, Boaden R, Hunter R, et al. Innovations in major system reconfiguration in England: a study of the effectiveness, acceptability and processes of implementation of two models of stroke care. *Implementation science : IS.* 2013;8:5.

56. Furler J, Young D, Best J, et al. Can primary care team-based transition to insulin improve outcomes in adults with type 2 diabetes: the stepping up to insulin cluster randomized controlled trial protocol. *Implementation Science.* 2014;9(1):20.

57. Gagliardi AR, Stuart-McEwan T, Gilbert J, et al. How can diagnostic assessment programs be implemented to enhance inter-professional collaborative care for cancer? *Implementation Science.* 2014;9(1):4.

58. Gama E, Madan J, Banda H, Squire B, Thomson R, Namakhoma I. Economic evaluation of the practical approach to lung health and informal provider interventions for improving the detection of tuberculosis and chronic airways disease at primary care level in Malawi: study protocol for cost-effectiveness analysis. *Implementation science : IS.* 2015;10:1.

59. Garner BR, Godley SH, Dennis ML, Godley MD, Shepard DS. The Reinforcing Therapist Performance (RTP) experiment: study protocol for a cluster randomized trial. *Implementation science : IS.* 2010;5:5.

60. Gattellari M, Leung DY, Ukoumunne OC, Zwar N, Grimshaw J, Worthington JM. Study protocol: the DESPATCH study: Delivering stroke prevention for patients with atrial fibrillation-a cluster randomised controlled trial in primary healthcare. *Implementation science : IS.* 2011;6:48.

61. Gattellari M, Worthington JM, Leung DY, Zwar N. Supporting Treatment decision making to Optimise the Prevention of STROKE in Atrial Fibrillation: The STOP STROKE in AF study. Protocol for a cluster randomised controlled trial. *Implementation Science.* 2012;7(1):63.

62. Gifford WA, Davies B, Graham ID, Lefebre N, Tourangeau A, Woodend K. A Quantitative and Qualitative methods pilot study with a cluster randomized control trial to evaluate the impact of a leadership intervention on guideline implementation in home care nursing. *Implementation science : IS.* 2008;3:51.

63. Gold R, Hollombe C, Bunce A, et al. Study protocol for "Study of Practices Enabling Implementation and Adaptation in the Safety Net (SPREAD-NET)": a pragmatic trial comparing implementation strategies. *Implementation science : IS.* 2015;10:144.

64. Gong W, Xu D, Zhou L, Brown HS, 3rd, Smith KL, Xiao S. Village doctor-assisted case management of rural patients with schizophrenia: protocol for a cluster randomized control trial. *Implementation science : IS.* 2014;9:13.

65. Green SE, Bosch M, McKenzie JE, et al. Improving the care of people with traumatic brain injury through the Neurotrauma Evidence Translation (NET) program: protocol for a program of research. *Implementation science : IS.* 2012;7:74.

66. Gude WT, Roos-Blom MJ, van der Veer SN, et al. Electronic audit and feedback intervention with action implementation toolbox to improve pain management in intensive care: protocol for a laboratory experiment and cluster randomised trial. *Implementation science : IS.* 2017;12(1):68.

67. Hack TF, Ruether JD, Weir LM, Grenier D, Degner LF. Study protocol: addressing evidence and context to facilitate transfer and uptake of consultation recording use in oncology: a knowledge translation implementation study. *Implementation science : IS.* 2011;6:20.

68. Hagedorn HJ, Brown R, Dawes M, et al. Enhancing access to alcohol use disorder pharmacotherapy and treatment in primary care settings: ADaPT-PC. *Implementation science : IS.* 2016;11:64.

69. Hall L, Farrington A, Mitchell BG, et al. Researching effective approaches to cleaning in hospitals: protocol of the REACH study, a multi-site stepped-wedge randomised trial. *Implementation science : IS.* 2016;11:44.

70. Hamilton AB, Mittman BS, Williams JK, et al. Community-based implementation and effectiveness in a randomized trial of a risk reduction intervention for HIV-serodiscordant couples: study protocol. *Implementation science : IS.* 2014;9:79.

71. Hanbury A, Thompson C, Wilson PM, et al. Translating research into practice in Leeds and Bradford (TRiPLaB): a protocol for a programme of research. *Implementation science : IS.* 2010;5:37.

72. Hanson C, Waiswa P, Marchant T, et al. Expanded Quality Management Using Information Power (EQUIP): protocol for a quasi-experimental study to improve maternal and newborn health in Tanzania and Uganda. *Implementation science : IS.* 2014;9(1):41.

73. Hanson C, Waiswa P, Marchant T, et al. Erratum to: Expanded Quality Management Using Information Power (EQUIP): protocol for a quasi-experimental study to improve maternal and newborn health in Tanzania and Uganda. *Implementation science : IS.* 2015;10:152.

74. Harris MF, Lloyd J, Litt J, et al. Preventive evidence into practice (PEP) study: implementation of guidelines to prevent primary vascular disease in general practice protocol for a cluster randomised controlled trial. *Implementation science : IS.* 2013;8(8).

75. Hartley S, Foy R, Walwyn REA, et al. The evaluation of enhanced feedback interventions to reduce unnecessary blood transfusions (AFFINITIE): protocol for two linked cluster randomised factorial controlled trials. *Implementation science : IS.* 2017;12(1):84.

76. Hartzler B, Lyon AR, Walker DD, Matthews L, King KM, McCollister KE. Implementing the teen marijuana check-up in schools-a study protocol. *Implementation science : IS.* 2017;12(1):103.

77. Hayek A, Joshi R, Usherwood T, et al. An integrated general practice and pharmacy-based intervention to promote the use of appropriate preventive medications among individuals at high cardiovascular disease risk: protocol for a cluster randomized controlled trial. *Implementation science : IS.* 2016;11(1):129.

78. Herschell AD, Kolko DJ, Scudder AT, et al. Protocol for a statewide randomized controlled trial to compare three training models for implementing an evidence-based treatment. *Implementation science : IS.* 2015;10:133.

79. Heselmans A, Van de Velde S, Ramaekers D, Vander Stichele R, Aertgeerts B. Feasibility and impact of an evidence-based electronic decision support system for diabetes care in family medicine: protocol for a cluster randomized controlled trial. *Implementation science : IS.* 2013;8:83.

80. Highfield L, Rajan SS, Valerio MA, Walton G, Fernandez ME, Bartholomew LK. A non-randomized controlled stepped wedge trial to evaluate the effectiveness of a multi-level mammography intervention in improving appointment adherence in underserved women. *Implementation science : IS.* 2015;10:143.

81. Hocking JS, Temple-Smith M, van Driel M, et al. Can preventive care activities in general practice be sustained when financial incentives and external audit plus feedback are removed? ACCEPt-able: a cluster randomised controlled trial protocol. *Implementation science : IS.* 2016;11(1):122.

82. Houston TK, Sadasivam RS, Ford DE, Richman J, Ray MN, Allison JJ. The QUIT-PRIMO provider-patient Internet-delivered smoking cessation referral intervention: a cluster-randomized comparative effectiveness trial: study protocol. *Implementation science : IS.* 2010;5:87.

83. Huis A, Schoonhoven L, Grol R, et al. Helping hands: a cluster randomised trial to evaluate the effectiveness of two different strategies for promoting hand hygiene in hospital nurses. *Implementation science : IS.* 2011;6:101.

84. Hunter SB, Ayer L, Han B, Garner BR, Godley SH. Examining the sustainment of the Adolescent-Community Reinforcement Approach in community addiction treatment settings: protocol for a longitudinal Quantitative and Qualitative method study. *Implementation science : IS.* 2014;9:104.

85. Husebo BS, Flo E, Aarsland D, et al. COSMOS--improving the quality of life in nursing home patients: protocol for an effectiveness-implementation cluster randomized clinical hybrid trial. *Implementation science : IS.* 2015;10:131.

86. Hutchinson AM, Sales AE, Brotto V, Bucknall TK. Implementation of an audit with feedback knowledge translation intervention to promote medication error reporting in health care: a protocol. *Implementation science : IS.* 2015;10:70.

87. Imms C, Novak I, Kerr C, et al. Improving allied health professionals' research implementation behaviours for children with cerebral palsy: protocol for a before-after study. *Implementation science : IS.* 2015;10:16.

88. Ista E, Trogrlic Z, Bakker J, Osse RJ, van Achterberg T, van der Jagt M. Improvement of care for ICU patients with delirium by early screening and treatment: study protocol of iDECePTIvE study. *Implementation science : IS.* 2014;9:143.

89. Ivers NM, Tu K, Francis J, et al. Feedback GAP: study protocol for a cluster-randomized trial of goal setting and action plans to increase the effectiveness of audit and feedback interventions in primary care. *Implementation science : IS.* 2010;5:98.

90. Ivers NM, Schwalm JD, Grimshaw JM, et al. Delayed educational reminders for long-term medication adherence in ST-elevation myocardial infarction (DERLA-STEMI): protocol for a pragmatic, cluster-randomized controlled trial. *Implementation science : IS.* 2012;7:54.

91. Ivers NM, Desveaux L, Presseau J, et al. Testing feedback message framing and comparators to address prescribing of high-risk medications in nursing homes: protocol for a pragmatic, factorial, cluster-randomized trial. *Implementation science : IS.* 2017;12(1):86.

92. Jabbour M, Curran J, Scott SD, et al. Best strategies to implement clinical pathways in an emergency department setting: study protocol for a cluster randomized controlled trial. *Implementation science : IS.* 2013;8:55.

93. Jabbour M, Reid S, Polihronis C, et al. Improving mental health care transitions for children and youth: a protocol to implement and evaluate an emergency department clinical pathway. *Implementation science : IS.* 2016;11(1):90.

94. James AS, Richardson V, Wang JS, Proctor EK, Colditz GA. Systems intervention to promote colon cancer screening in safety net settings: protocol for a community-based participatory randomized controlled trial. *Implementation science : IS.* 2013;8:58.

95. Jensen CE, Riis A, Pedersen KM, Jensen MB, Petersen KD. Study protocol of an economic evaluation of an extended implementation strategy for the treatment of low back pain in general practice: a cluster randomised controlled trial. *Implementation science : IS.* 2014;9:140.

96. Jeon YH, Simpson JM, Chenoweth L, Cunich M, Kendig H. The effectiveness of an aged care specific leadership and management program on workforce, work environment, and care quality outcomes: design of a cluster randomised controlled trial. *Implementation science : IS.* 2013;8:126.

97. Johnson DW, Craig W, Brant R, Mitton C, Svenson L, Klassen TP. A cluster randomized controlled trial comparing three methods of disseminating practice guidelines for children with croup [ISRCTN73394937]. *Implementation science : IS.* 2006;1:10.

98. Jsbrandy CI, Ottevanger PB, Groen WG, Gerritsen WR, van Harten WH, Hermens RP. Study protocol: an evaluation of the effectiveness, experiences and costs of a patient-directed strategy compared with a multi-faceted strategy to implement physical cancer rehabilitation programmes for cancer survivors in a European healthcare system; a controlled before and after study. *Implementation science : IS.* 2015;10:128.

99. Kennedy CC, Ioannidis G, Giangregorio LM, et al. An interdisciplinary knowledge translation intervention in long-term care: study protocol for the vitamin D and osteoporosis study (ViDOS) pilot cluster randomized controlled trial. *Implementation science : IS.* 2012;7:48.

100. Keriel-Gascou M, Buchet-Poyau K, Duclos A, et al. Evaluation of an interactive program for preventing adverse drug events in primary care: study protocol of the InPAct cluster randomised stepped wedge trial. *Implementation Science.* 2013;8(1):69.

101. Keurhorst MN, Anderson P, Spak F, et al. Implementing training and support, financial reimbursement, and referral to an internet-based brief advice program to improve the early identification of hazardous and harmful alcohol consumption in primary care (ODHIN): study protocol for a cluster randomized factorial trial. *Implementation science : IS.* 2013;8:11.

102. Kilbourne AM, Almirall D, Eisenberg D, et al. Protocol: Adaptive Implementation of Effective Programs Trial (ADEPT): cluster randomized SMART trial comparing a standard versus enhanced implementation strategy to improve outcomes of a mood disorders program. *Implementation science : IS.* 2014;9:132.

103. Knight DK, Belenko S, Wiley T, et al. Juvenile Justice-Translational Research on Interventions for Adolescents in the Legal System (JJ-TRIALS): a cluster randomized trial targeting system-wide improvement in substance use services. *Implementation science : IS.* 2016;11:57.

104. Krist AH, Aycock RA, Etz RS, et al. MyPreventiveCare: implementation and dissemination of an interactive preventive health record in three practice-based research networks serving disadvantaged patients--a randomized cluster trial. *Implementation science : IS.* 2014;9:181.

105. Lavis JN, Wilson MG, Grimshaw JM, et al. Effects of an evidence service on health-system policy makers' use of research evidence: a protocol for a randomised controlled trial. *Implementation science : IS.* 2011;6:51.

106. Lee TM, Ivers NM, Bhatia S, et al. Improving stroke prevention therapy for patients with atrial fibrillation in primary care: protocol for a pragmatic, cluster-randomized trial. *Implementation science : IS.* 2016;11(1):159.

107. Lewis CC, Scott K, Marti CN, et al. Implementing measurement-based care (iMBC) for depression in community mental health: a dynamic cluster randomized trial study protocol. *Implementation science : IS.* 2015;10:127.

108. Li F, Harmer P. Protocol for disseminating an evidence-based fall prevention program in community senior centers: evaluation of translatability and public health impact via a single group pre-post study. *Implementation science : IS.* 2014;9:63.

109. Liddy C, Hogg W, Russell G, et al. Improved delivery of cardiovascular care (IDOCC) through outreach facilitation: study protocol and implementation details of a cluster randomized controlled trial in primary care. *Implementation science : IS.* 2011;6:110.

110. Liu B, Almaawiy U, Moore JE, Chan WH, Straus SE, Team MO. Evaluation of a multisite educational intervention to improve mobilization of older patients in hospital: protocol for mobilization of vulnerable elders in Ontario (MOVE ON). *Implementation science : IS.* 2013;8:76.

111. Lubinga SJ, Jenny AM, Larsen-Cooper E, et al. Impact of pharmacy worker training and deployment on access to essential medicines and health outcomes in Malawi: protocol for a cluster quasi-experimental evaluation. *Implementation science : IS.* 2014;9:156.

112. Luitjes SH, Wouters MG, Franx A, et al. Study protocol: Cost effectiveness of two strategies to implement the NVOG guidelines on hypertension in pregnancy: An innovative strategy including a computerised decision support system compared to a common strategy of professional audit and feedback, a randomized controlled trial. *Implementation science : IS.* 2010;5:68.

113. MacDermid JC, Solomon P, Law M, Russell D, Stratford P. Defining the effect and mediators of two knowledge translation strategies designed to alter knowledge, intent and clinical utilization of rehabilitation outcome measures: a study protocol [NCT00298727]. *Implementation science : IS.* 2006;1:14.

114. MacDermid JC, Law M, Buckley N, Haynes RB. "Push" versus "Pull" for mobilizing pain evidence into practice across different health professions: a protocol for a randomized trial. *Implementation science : IS.* 2012;7:115.

115. Mann DM, Kannry JL, Edonyabo D, et al. Rationale, design, and implementation protocol of an electronic health record integrated clinical prediction rule (iCPR) randomized trial in primary care. *Implementation science : IS.* 2011;6:109.

116. Mann DM, Lin JJ. Increasing efficacy of primary care-based counseling for diabetes prevention: rationale and design of the ADAPT (Avoiding Diabetes Thru Action Plan Targeting) trial. *Implementation science : IS.* 2012;7:6.

117. Martino S, Zimbrean P, Forray A, et al. See One, Do One, Order One: a study protocol for cluster randomized controlled trial testing three strategies for implementing motivational interviewing on medical inpatient units. *Implementation science : IS.* 2015;10:138.

118. McAlister FA, Fradette M, Graham M, et al. A randomized trial to assess the impact of opinion leader endorsed evidence summaries on the use of secondary prevention strategies in patients with coronary artery disease: the ESP-CAD trial protocol [NCT00175240]. *Implementation science : IS.* 2006;1:11.

119. McEwen SE, Donald M, Dawson D, et al. A multi-faceted knowledge translation approach to support persons with stroke and cognitive impairment: evaluation protocol. *Implementation science : IS.* 2015;10:157.

120. McKenzie JE, French SD, O'Connor DA, et al. IMPLEmenting a clinical practice guideline for acute low back pain evidence-based manageMENT in general practice (IMPLEMENT): cluster randomised controlled trial study protocol. *Implementation science : IS.* 2008;3:11.

121. McKenzie JE, O'Connor DA, Page MJ, et al. Improving the care for people with acute low-back pain by allied health professionals (the ALIGN trial): A cluster randomised trial protocol. *Implementation science : IS.* 2010;5:86.

122. McKenzie JE, French SD, O'Connor DA, et al. Evidence-based care of older people with suspected cognitive impairment in general practice: protocol for the IRIS cluster randomised trial. *Implementation science : IS.* 2013;8:91.

123. Melman S, Schoorel EN, Dirksen C, et al. SIMPLE: implementation of recommendations from international evidence-based guidelines on caesarean sections in the Netherlands. Protocol for a controlled before and after study. *Implementation science : IS.* 2013;8:3.

124. Menya D, Logedi J, Manji I, Armstrong J, Neelon B, O'Meara WP. An innovative pay-for-performance (P4P) strategy for improving malaria management in rural Kenya: protocol for a cluster randomized controlled trial. *Implementation science : IS.* 2013;8:48.

125. Mettes TG, van der Sanden WJ, Wensing M, Grol RP, Plasschaert AJ. A cluster randomised controlled trial in primary dental care based intervention to improve professional performance on routine oral examinations and the management of asymptomatic impacted third molars: study protocol. *Implementation science : IS.* 2007;2:12.

126. Middleton S, Levi C, Ward J, et al. Fever, hyperglycaemia and swallowing dysfunction management in acute stroke: A cluster randomised controlled trial of knowledge transfer. *Implementation Science.* 2009;4(1):16.

127. Middleton S, Levi C, Dale S, et al. Triage, treatment and transfer of patients with stroke in emergency department trial (the T3 Trial): a cluster randomised trial protocol. *Implementation science : IS.* 2016;11(1):139.

128. Minian N, Baliunas D, Zawertailo L, et al. Combining alcohol interventions with tobacco addictions treatment in primary care-the COMBAT study: a pragmatic cluster randomized trial. *Implementation science : IS.* 2017;12(1):65.

129. Moja L, Moschetti I, Cinquini M, et al. Clinical evidence continuous medical education: a randomised educational trial of an open access e-learning program for transferring evidence-based information - ICEKUBE (Italian Clinical Evidence Knowledge Utilization Behaviour Evaluation) - study protocol. *Implementation science : IS.* 2008;3:37.

130. Moja L, Polo Friz H, Capobussi M, et al. Implementing an evidence-based computerized decision support system to improve patient care in a general hospital: the CODES study protocol for a randomized controlled trial. *Implementation science : IS.* 2016;11(1):89.

131. Moja L, Passardi A, Capobussi M, et al. Implementing an evidence-based computerized decision support system linked to electronic health records to improve care for cancer patients: the ONCO-CODES study protocol for a randomized controlled trial. *Implementation science : IS.* 2016;11(1):153.

132. Molfenter T, Kim JS, Quanbeck A, Patel-Porter T, Starr S, McCarty D. Testing use of payers to facilitate evidence-based practice adoption: protocol for a cluster-randomized trial. *Implementation science : IS.* 2013;8:50.

133. Mortimer D, French SD, McKenzie JE, O'Connor DA, Green SE, group Is. Protocol for economic evaluation alongside the IMPLEMENT cluster randomised controlled trial. *Implementation science : IS.* 2008;3:12.

134. Nouwens E, Van Lieshout J, Adang E, Bouma M, Braspenning J, Wensing M. Effectiveness and efficiency of a practice accreditation program on cardiovascular risk management in primary care: study protocol of a clustered randomized trial. *Implementation science : IS.* 2012;7:94.

135. Ober AJ, Watkins KE, Hunter SB, Lamp K, Lind M, Setodji CM. An organizational readiness intervention and randomized controlled trial to test strategies for implementing substance use disorder treatment into primary care: SUMMIT study protocol. *Implementation science : IS.* 2015;10:66.

136. Osteras N, van Bodegom-Vos L, Dziedzic K, et al. Implementing international osteoarthritis treatment guidelines in primary health care: study protocol for the SAMBA stepped wedge cluster randomized controlled trial. *Implementation science : IS.* 2015;10:165.

137. Ostroff JS, Li Y, Shelley DR. Dentists United to Extinguish Tobacco (DUET): a study protocol for a cluster randomized, controlled trial for enhancing implementation of clinical practice guidelines for treating tobacco dependence in dental care settings. *Implementation science : IS.* 2014;9:25.

138. Owen RR, Drummond KL, Viverito KM, et al. Monitoring and managing metabolic effects of antipsychotics: a cluster randomized trial of an intervention combining evidence-based quality improvement and external facilitation. *Implementation science : IS.* 2013;8:120.

139. Parchman ML, Pugh JA, Culler SD, et al. A group randomized trial of a complexity-based organizational intervention to improve risk factors for diabetes complications in primary care settings: study protocol. *Implementation science : IS.* 2008;3:15.

140. Parchman ML, Fagnan LJ, Dorr DA, et al. Study protocol for "Healthy Hearts Northwest": a 2 x 2 randomized factorial trial to build quality improvement capacity in primary care. *Implementation science : IS.* 2016;11(1):138.

141. Paul CL, Piterman L, Shaw J, et al. Diabetes in rural towns: effectiveness of continuing education and feedback for healthcare providers in altering diabetes outcomes at a population level: protocol for a cluster randomised controlled trial. *Implementation science : IS.* 2013;8:30.

142. Paul CL, Levi CR, D'Este CA, et al. Thrombolysis ImPlementation in Stroke (TIPS): evaluating the effectiveness of a strategy to increase the adoption of best evidence practice--protocol for a cluster randomised controlled trial in acute stroke care. *Implementation science : IS.* 2014;9:38.

143. Peiris D, Sun L, Patel A, et al. Systematic medical assessment, referral and treatment for diabetes care in China using lay family health promoters: protocol for the SMARTDiabetes cluster randomised controlled trial. *Implementation science : IS.* 2016;11(1):116.

144. Pellecchia M, Beidas RS, Marcus SC, et al. Study protocol: implementation of a computer-assisted intervention for autism in schools: a hybrid type II cluster randomized effectiveness-implementation trial. *Implementation science : IS.* 2016;11(1):154.

145. Petersen LA, Urech T, Simpson K, et al. Design, rationale, and baseline characteristics of a cluster randomized controlled trial of pay for performance for hypertension treatment: study protocol. *Implementation science : IS.* 2011;6:114.

146. Pinto D, Heleno B, Rodrigues DS, Papoila AL, Santos I, Caetano PA. An open cluster-randomized, 18-month trial to compare the effectiveness of educational outreach visits with usual guideline dissemination to improve family physician prescribing. *Implementation science : IS.* 2014;9:10.

147. Prados-Torres A, Del Cura-Gonzalez I, Prados-Torres D, et al. Effectiveness of an intervention for improving drug prescription in primary care patients with multimorbidity and polypharmacy: study protocol of a cluster randomized clinical trial (Multi-PAP project). *Implementation science : IS.* 2017;12(1):54.

148. Praveen D, Patel A, McMahon S, et al. A multifaceted strategy using mobile technology to assist rural primary healthcare doctors and frontline health workers in cardiovascular disease risk management: protocol for the SMARTHealth India cluster randomised controlled trial. *Implementation science : IS.* 2013;8:137.

149. Prior M, Elouafkaoui P, Elders A, et al. Evaluating an audit and feedback intervention for reducing antibiotic prescribing behaviour in general dental practice (the RAPiD trial): a partial factorial cluster randomised trial protocol. *Implementation science : IS.* 2014;9:50.

150. Pugh MJ, Leykum LK, Lanham HJ, et al. Implementation of the Epilepsy Center of Excellence to improve access to and quality of care--protocol for a Quantitative and Qualitative methods study. *Implementation science : IS.* 2014;9(1):44.

151. Quanbeck AR, Gustafson DH, Ford JH, 2nd, et al. Disseminating quality improvement: study protocol for a large cluster-randomized trial. *Implementation science : IS.* 2011;6:44.

152. Quanbeck AR, Gustafson DH, Marsch LA, et al. Integrating addiction treatment into primary care using mobile health technology: protocol for an implementation research study. *Implementation science : IS.* 2014;9:65.

153. Rabbani F, Mukhi AA, Perveen S, et al. Improving community case management of diarrhoea and pneumonia in district Badin, Pakistan through a cluster randomised study--the NIGRAAN trial protocol. *Implementation science : IS.* 2014;9:186.

154. Ramsey AT, Maki J, Prusaczyk B, Yan Y, Wang J, Lobb R. Using segmented regression analysis of interrupted time series data to assess colonoscopy quality outcomes of a web-enhanced implementation toolkit to support evidence-based practices for bowel preparation: a study protocol. *Implementation science : IS.* 2015;10:85.

155. Ranta A, Dovey S, Weatherall M, O'Dea D. Efficacy and safety of a TIA/stroke electronic support tool (FASTEST) trial: study protocol. *Implementation science : IS.* 2012;7:107.

156. Ridde V, Turcotte-Tremblay AM, Souares A, et al. Protocol for the process evaluation of interventions combining performance-based financing with health equity in Burkina Faso. *Implementation science : IS.* 2014;9:149.

157. Riis A, Jensen CE, Bro F, Maindal HT, Petersen KD, Jensen MB. Enhanced implementation of low back pain guidelines in general practice: study protocol of a cluster randomised controlled trial. *Implementation Science.* 2013;8(1):124.

158. Rosella L, Peirson L, Bornbaum C, et al. Supporting collaborative use of the Diabetes Population Risk Tool (DPoRT) in health-related practice: a multiple case study research protocol. *Implementation science : IS.* 2014;9(1):35.

159. Sales AE, Ersek M, Intrator OK, et al. Implementing goals of care conversations with veterans in VA long-term care setting: a Quantitative and Qualitative methods protocol. *Implementation science : IS.* 2016;11(1):132.

160. Scales DC, Dainty K, Hales B, et al. An innovative telemedicine knowledge translation program to improve quality of care in intensive care units: protocol for a cluster randomized pragmatic trial. *Implementation science : IS.* 2009;4:5.

161. Scott S, Hartling L, Grimshaw J, et al. Improving outcomes for ill and injured children in emergency departments: protocol for a program in pediatric emergency medicine and knowledge translation science. *Implementation science : IS.* 2009;4:60.

162. Seers K, Cox K, Crichton NJ, et al. FIRE (Facilitating Implementation of Research Evidence): a study protocol. *Implementation science : IS.* 2012;7:25.

163. Shelley D, VanDevanter N, Cleland CC, Nguyen L, Nguyen N. Implementing tobacco use treatment guidelines in community health centers in Vietnam. *Implementation science : IS.* 2015;10:142.

164. Shelley DR, Ogedegbe G, Anane S, et al. Testing the use of practice facilitation in a cluster randomized stepped-wedge design trial to improve adherence to cardiovascular disease prevention guidelines: HealthyHearts NYC. *Implementation science : IS.* 2016;11(1):88.

165. Sherr K, Gimbel S, Rustagi A, et al. Systems analysis and improvement to optimize pMTCT (SAIA): a cluster randomized trial. *Implementation science : IS.* 2014;9:55.

166. Siebenhofer A, Ulrich LR, Mergenthal K, et al. Primary care management for optimized antithrombotic treatment [PICANT]: study protocol for a cluster-randomized controlled trial. *Implementation science : IS.* 2012;7:79.

167. Simmons MM, Gabrielian S, Byrne T, et al. A Hybrid III stepped wedge cluster randomized trial testing an implementation strategy to facilitate the use of an evidence-based practice in VA Homeless Primary Care Treatment Programs. *Implementation science : IS.* 2017;12(1):46.

168. Sinnema H, Franx G, Volker D, et al. Randomised controlled trial of tailored interventions to improve the management of anxiety and depressive disorders in primary care. *Implementation science : IS.* 2011;6:75.

169. Smelson DA, Chinman M, McCarthy S, Hannah G, Sawh L, Glickman M. A cluster randomized Hybrid Type III trial testing an implementation support strategy to facilitate the use of an evidence-based practice in VA homeless programs. *Implementation science : IS.* 2015;10:79.

170. Smith SA, Blumenthal DS. Efficacy to effectiveness transition of an Educational Program to Increase Colorectal Cancer Screening (EPICS): study protocol of a cluster randomized controlled trial. *Implementation science : IS.* 2013;8:86.

171. Spaulding A, Fendrick AM, Herman WH, et al. A controlled trial of value-based insurance design - the MHealthy: Focus on Diabetes (FOD) trial. *Implementation science : IS.* 2009;4:19.

172. Squires JE, Suh KN, Linklater S, et al. Improving physician hand hygiene compliance using behavioural theories: a study protocol. *Implementation science : IS.* 2013;8:16.

173. Squires JE, Grimshaw JM, Taljaard M, et al. Design, implementation, and evaluation of a knowledge translation intervention to increase organ donation after cardiocirculatory death in Canada: a study protocol. *Implementation science : IS.* 2014;9:80.

174. Stacey D, Bakker D, Ballantyne B, et al. Managing symptoms during cancer treatments: evaluating the implementation of evidence-informed remote support protocols. *Implementation science : IS.* 2012;7:110.

175. Stacey D, Taljaard M, Smylie J, et al. Implementation of a patient decision aid for men with localized prostate cancer: evaluation of patient outcomes and practice variation. *Implementation science : IS.* 2016;11(1):87.

176. Staedke SG, Chandler CI, DiLiberto D, et al. The PRIME trial protocol: evaluating the impact of an intervention implemented in public health centres on management of malaria and health outcomes of children using a cluster-randomised design in Tororo, Uganda. *Implementation science : IS.* 2013;8:114.

177. Stiell IG, Grimshaw J, Wells GA, et al. A matched-pair cluster design study protocol to evaluate implementation of the Canadian C-spine rule in hospital emergency departments: Phase III. *Implementation science : IS.* 2007;2:4.

178. Stienen JJ, Hermens RP, Wennekes L, et al. Improvement of hospital care for patients with non-Hodgkin's lymphoma: protocol for a cluster randomized controlled trial (PEARL study). *Implementation science : IS.* 2013;8:77.

179. Stirman SW, Shields N, Deloriea J, et al. A randomized controlled dismantling trial of post-workshop consultation strategies to increase effectiveness and fidelity to an evidence-based psychotherapy for Posttraumatic stress disorder. *Implementation Science.* 2013;8(1):82.

180. Swindle T, Johnson SL, Whiteside-Mansell L, Curran GM. A Quantitative and Qualitative methods protocol for developing and testing implementation strategies for evidence-based obesity prevention in childcare: a cluster randomized hybrid type III trial. *Implementation science : IS.* 2017;12(1):90.

181. Tapp H, McWilliams A, Ludden T, et al. Comparing traditional and participatory dissemination of a shared decision making intervention (ADAPT-NC): a cluster randomized trial. *Implementation science : IS.* 2014;9:158.

182. te Boveldt N, Engels Y, Besse K, Vissers K, Vernooij-Dassen M. Rationale, design, and implementation protocol of the Dutch clinical practice guideline pain in patients with cancer: a cluster randomised controlled trial with Short Message Service (SMS) and Interactive Voice Response (IVR). *Implementation science : IS.* 2011;6:126.

183. Tello-Bernabe E, Sanz-Cuesta T, del Cura-Gonzalez I, et al. Effectiveness of a clinical practice guideline implementation strategy for patients with anxiety disorders in primary care: cluster randomized trial. *Implementation Science.* 2011;6(123):2-9.

184. Toftegaard BS, Bro F, Vedsted P. A geographical cluster randomised stepped wedge study of continuing medical education and cancer diagnosis in general practice. *Implementation science : IS.* 2014;9:159.

185. Trautner BW, Kelly PA, Petersen N, et al. A hospital-site controlled intervention using audit and feedback to implement guidelines concerning inappropriate treatment of catheter-associated asymptomatic bacteriuria. *Implementation science : IS.* 2011;6:41.

186. Tremblay D, Drouin D, Lang A, Roberge D, Ritchie J, Plante A. Interprofessional collaborative practice within cancer teams: Translating evidence into action. A Quantitative and Qualitative methods study protocol. *Implementation science : IS.* 2010;5:53.

187. Treweek S, Ricketts IW, Francis J, et al. Developing and evaluating interventions to reduce inappropriate prescribing by general practitioners of antibiotics for upper respiratory tract infections: a randomised controlled trial to compare paper-based and web-based modelling experiments. *Implementation science : IS.* 2011;6:16.

188. Trietsch J, van der Weijden T, Verstappen W, et al. A cluster randomized controlled trial aimed at implementation of local quality improvement collaboratives to improve prescribing and test ordering performance of general practitioners: study protocol. *Implementation science : IS.* 2009;4:6.

189. Van de Steeg L, Langelaan M, Ijkema R, Wagner C. The effect of a complementary e-learning course on implementation of a quality improvement project regarding care for elderly patients: a stepped wedge trial. *Implementation science : IS.* 2012;7:13.

190. van den Boogaard NM, Kersten FA, Goddijn Mt, et al. Improving the implementation of tailored expectant management in subfertile couples: protocol for a cluster randomized trial. *Implementation science : IS.* 2013;8:53.

191. van Engen-Verheul MM, de Keizer NF, van der Veer SN, et al. Evaluating the effect of a web-based quality improvement system with feedback and outreach visits on guideline concordance in the field of cardiac rehabilitation: rationale and study protocol. *Implementation science : IS.* 2014;9:780.

192. van Gaalen JL, Bakker MJ, van Bodegom-Vos L, et al. Implementation strategies of internet-based asthma self-management support in usual care. Study protocol for the IMPASSE cluster randomized trial. *Implementation Science.* 2012;7(1):113.

193. van Lieshout J, Steenkamer B, Knippenberg M, Wensing M. Improvement of primary care for patients with chronic heart failure: A study protocol for a cluster randomised trial comparing two strategies. *Implementation science : IS.* 2011;6:28.

194. Voorn VM, Marang-van de Mheen PJ, So-Osman C, et al. De-implementation of expensive blood saving measures in hip and knee arthroplasties: study protocol for the LISBOA-II cluster randomized trial. *Implementation science : IS.* 2014;9:48.

195. Waldorff FB, Siersma V, Ertmann R, et al. The efficacy of computer reminders on external quality assessment for point-of-care testing in Danish general practice: rationale and methodology for two randomized trials. *Implementation science : IS.* 2011;6:79.

196. Watson DP, Young J, Ahonen E, et al. Development and testing of an implementation strategy for a complex housing intervention: protocol for a Quantitative and Qualitative methods study. *Implementation science : IS.* 2014;9:138.

197. Weiner BJ, Pignone MP, DuBard CA, et al. Advancing heart health in North Carolina primary care: the Heart Health NOW study protocol. *Implementation science : IS.* 2015;10:160.

198. Wensing M, Ludt S, Campbell S, et al. European Practice Assessment of Cardiovascular risk management (EPA Cardio): protocol of an international observational study in primary care. *Implementation science : IS.* 2009;4:3.

199. Willging CE, Green AE, Ramos MM. Implementing school nursing strategies to reduce LGBTQ adolescent suicide: a randomized cluster trial study protocol. *Implementation science : IS.* 2016;11(1):145.

200. Williams V, Oades LG, Deane FP, Crowe TP, Ciarrochi J, Andresen R. Improving implementation of evidence-based practice in mental health service delivery: protocol for a cluster randomised quasi-experimental investigation of staff-focused values interventions. *Implementation science : IS.* 2013;8:75.

201. Willis TA, Hartley S, Glidewell L, et al. Action to Support Practices Implement Research Evidence (ASPIRE): protocol for a cluster-randomised evaluation of adaptable implementation packages targeting 'high impact' clinical practice recommendations in general practice. *Implementation science : IS.* 2016;11:25.

202. Wilson MG, Lavis JN, Grimshaw JM, Haynes RB, Bekele T, Rourke SB. Effects of an evidence service on community-based AIDS service organizations' use of research evidence: a protocol for a randomized controlled trial. *Implementation science : IS.* 2011;6:52.

203. Wilson PM, Farley K, Thompson C, et al. Effects of a demand-led evidence briefing service on the uptake and use of research evidence by commissioners of health services: protocol for a controlled before and after study. *Implementation science : IS.* 2015;10:7.

204. Wiltsey Stirman S, Finley EP, Shields N, et al. Improving and sustaining delivery of CPT for PTSD in mental health systems: a cluster randomized trial. *Implementation science : IS.* 2017;12(1):32.

205. Wolfenden L, Nathan N, Williams CM, et al. A randomised controlled trial of an intervention to increase the implementation of a healthy canteen policy in Australian primary schools: study protocol. *Implementation science : IS.* 2014;9:147.

206. Wu RR, Myers RA, McCarty CA, et al. Protocol for the "Implementation, adoption, and utility of family history in diverse care settings" study. *Implementation science : IS.* 2015;10:163.

207. Yano EM, Darling JE, Hamilton AB, et al. Cluster randomized trial of a multilevel evidence-based quality improvement approach to tailoring VA Patient Aligned Care Teams to the needs of women Veterans. *Implementation science : IS.* 2016;11(1):101.

208. Zatzick DF, Russo J, Darnell D, et al. An effectiveness-implementation hybrid trial study protocol targeting posttraumatic stress disorder and comorbidity. *Implementation science : IS.* 2016;11:58.

209. Zwarenstein M, Hux JE, Kelsall D, et al. The Ontario printed educational message (OPEM) trial to narrow the evidence-practice gap with respect to prescribing practices of general and family physicians: a cluster randomized controlled trial, targeting the care of individuals with diabetes and hypertension in Ontario, Canada. *Implementation science : IS.* 2007;2:37.
