# Supplementary material for: Variation in Research Designs Used to Test the Effectiveness of Dissemination and Implementation Strategies: A Review
Source: Front Public Health. 2018 Feb 19;6:32. doi: 10.3389/fpubh.2018.00032 (PMC5826311; doi:10.3389/fpubh.2018.00032)
Supplement: Supplementary file 2 [file Data_Sheet_2.PDF]

Additional File 2. Hypothetical design choice scenarios

Scenario 1: Stepped wedge cluster randomized controlled trial

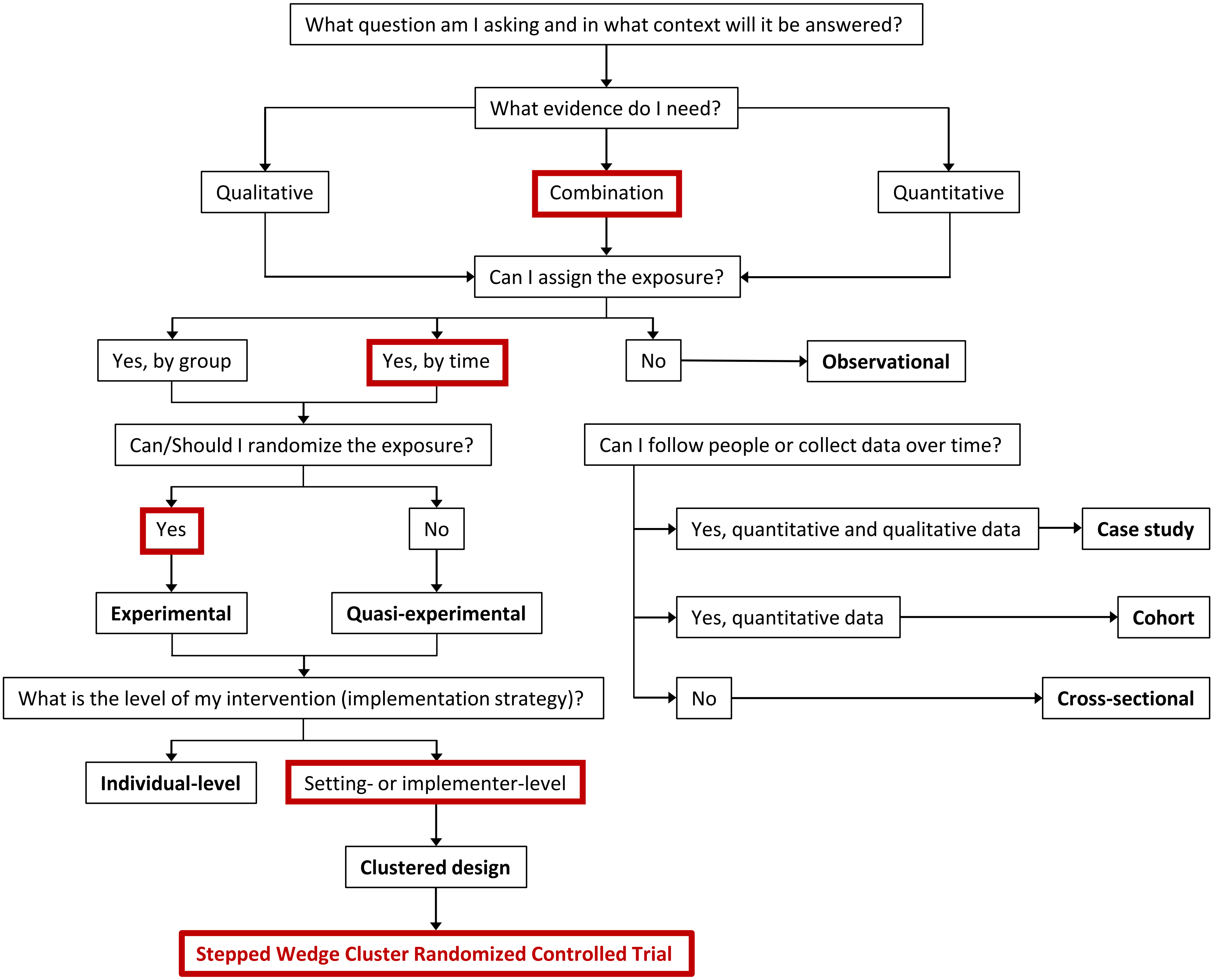

Researchers focused on nutrition of young children attending center-based care were interested in studying the implementation of an evidence-based intervention (EBI) focused on the improvement of foods and beverages served to preschool children while in child care and its impact on a center's fidelity to the recommendation to not serve preschoolers sugar-sweetened beverages (including non-100% fruit juice). The EBI was supported by train-the-trainer sessions (implementation strategy 1) conducted by research staff with technical assistance staff available to center directors, and educational toolkits for center directors and classroom teachers on how to implement the EBI (implementation strategy 2). Both quantitative data on foods served by the child care center to child and qualitative data changes to the organizational context of the child care center were of interest, and random assignment of the exposure was possible. These changes were made within a child care setting, so a cluster design was necessary. To manage budget/personnel constraints of the research staff and to account for any secular changes in foods served, a stepped wedge cluster randomized controlled trial was selected.

Scenario 2: Cluster randomized SMART implementation trial

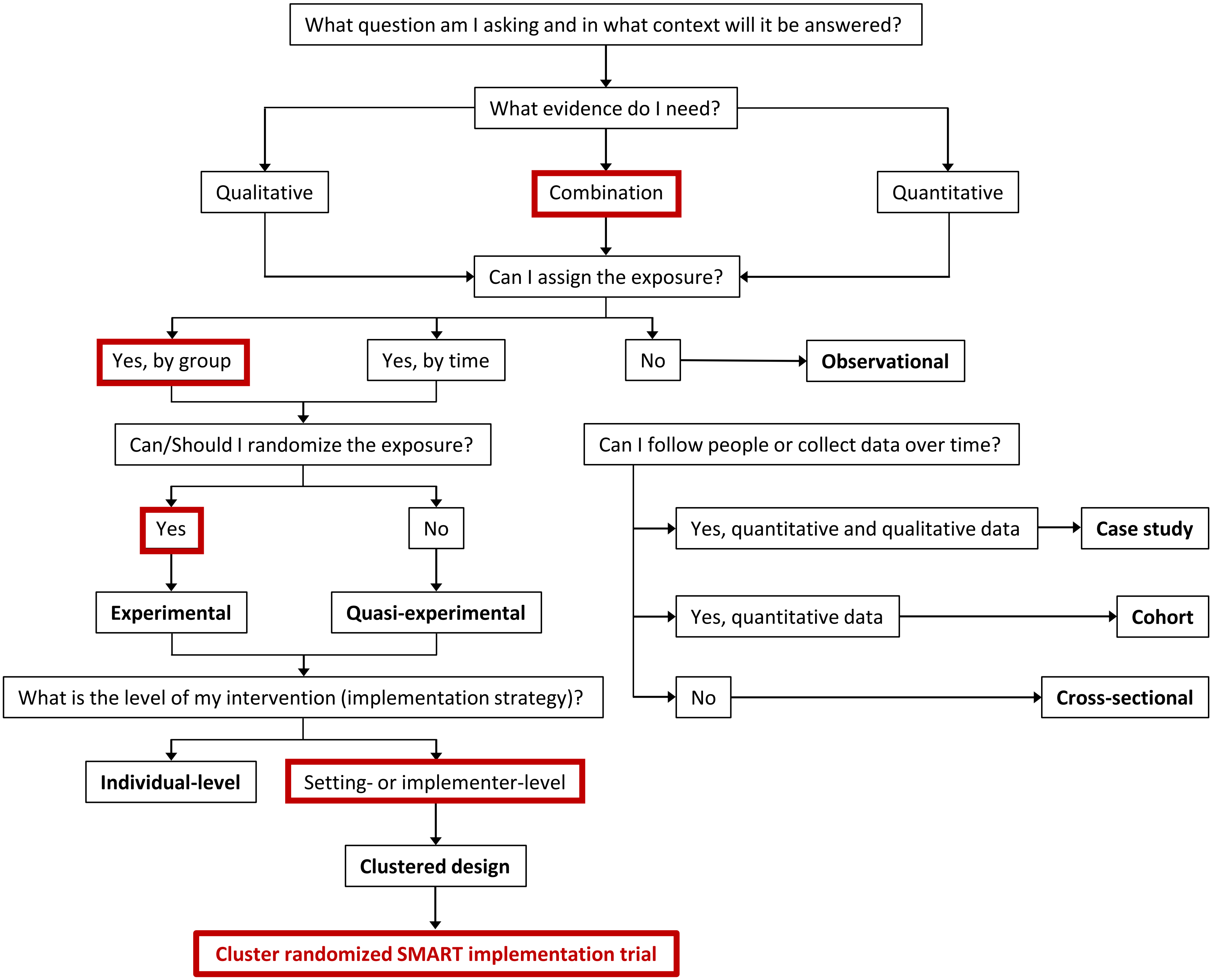

Researchers were interested in studying the supports needed to implement a new evidence-based program for simplification of drug treatment for older adults with multiple chronic diseases, particularly what sequence/combination of external supports were needed by hospital staff. Randomization was possible, and the active study implementation period was scheduled for 1 year (i.e., long enough to do multiple rounds of randomization). Thus, a cluster randomized implementation trial was selected. First, hospitals were randomized to receive audit and feedback (AF) on performance for the first 6 months or no external implementation support. Hospitals were deemed “non-responders” if at the end of the 6 months, they meeting less than 50% of the new guidelines. Hospital staff at non-responder hospitals were randomized to receive technical assistance from local specialists for 6 months or continue as originally randomized. Researchers wanted information via medical records on appropriateness of prescribing practices at the hospital level, and via in-depth interviews on how the AF information was used to support implementation, thus a combination of qualitative and quantitative data was used.

Scenario 3: Stepped wedge cluster randomized controlled trial

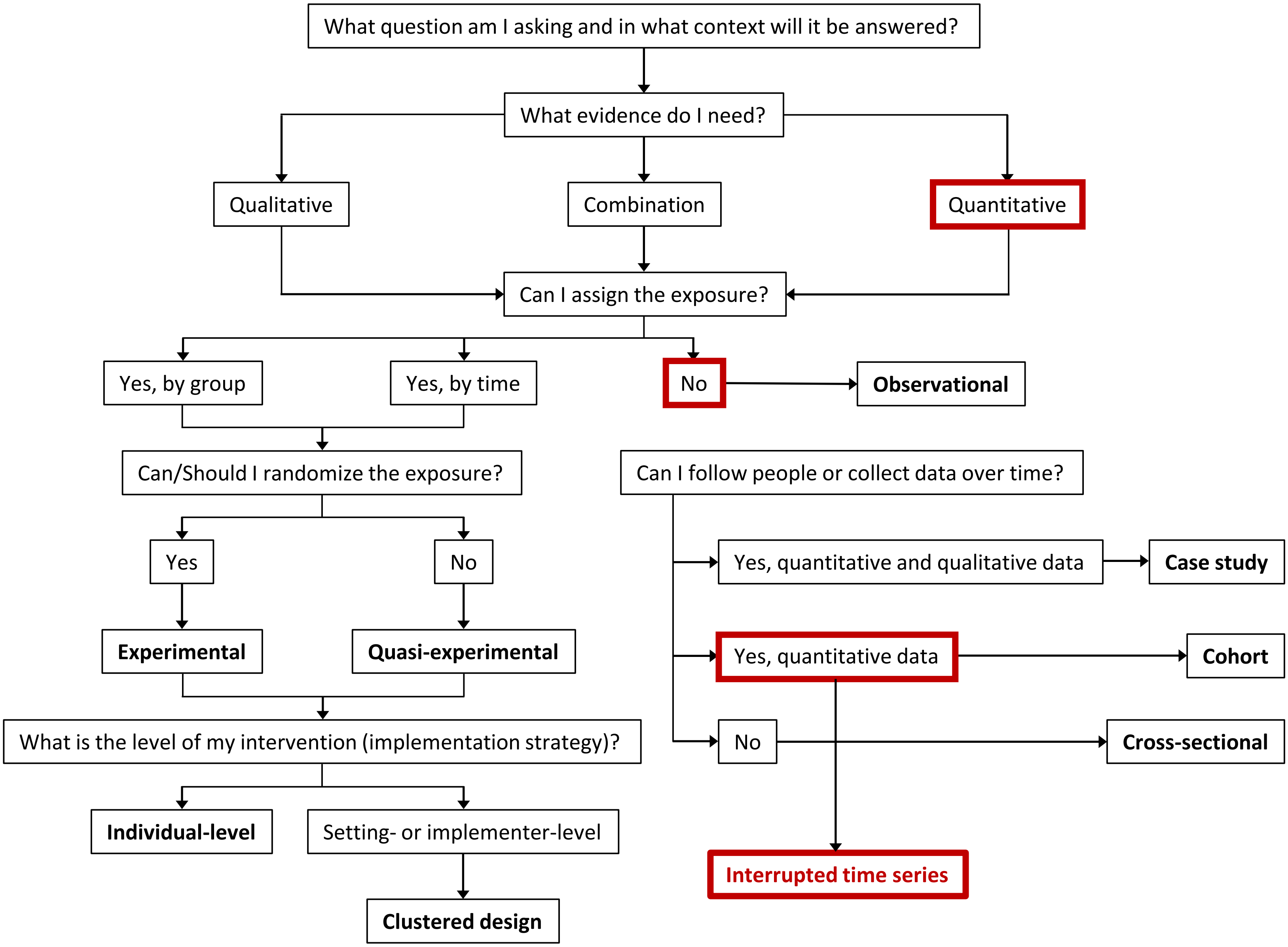

Researchers were interested studying the dissemination of a national policy mandating the presence of quitline information on electronic cigarette advertisements, measured by observed adherence to the mandate. No assignment was allowed because it was a national mandate; thus, an observational design was needed, and a comparison group was impractical. Vendors were given 18 months to comply with the policy, so it was possible to collect several time points of data before and after the mandate went into place. Thus, an interrupted time series design was chosen.
