# Supplementary material for: Variation in Research Designs Used to Test the Effectiveness of Dissemination and Implementation Strategies: A Review
Source: Front Public Health. 2018 Feb 19;6:32. doi: 10.3389/fpubh.2018.00032 (PMC5826311; doi:10.3389/fpubh.2018.00032)
Supplement: Supplementary file 3 [file Data_Sheet_3.PDF]

Additional File 3. Resources for additional guidance on design choice and D&I research

Washington University in St. Louis Dissemination and Implementation Research Core

- Dissemination and Implementation Research Toolkits (see "Designs" toolkit)

Brown et al. An Overview of Research and Evaluation Designs for Dissemination and Implementation. *Annual Review of Public Health.* 2017.

Landsverk J, Brown CH, Smith JD, et al. Design and Analysis in Dissemination and Implementation Research. In: Brownson RC, Colditz GA, Proctor EK, eds. Dissemination and Implementation Research in Health: Translating Science to Practice. 2nd ed. New York: Oxford University Press; 2017.

Implementation Science Webinar Series created by National Cancer Institute (NCI) Division of Cancer Control & Population Sciences Implementation Science Team

2014 Training Institute for Dissemination and Implementation Research in Health (TIDIRH) archive of presentations (webinars and PowerPoint slides)

- Designs for D&I Research (Dr. David Marrero)

Directory of Implementation Science Training Programs created by OSSR NIH

Penn State Methodology Center – resources for MOST, SMART, adaptive interventions, and factorial designs
